# Identification of small RNAs associated with RNA chaperone Hfq reveals a new stress response regulator in *Actinobacillus pleuropneumoniae*

**DOI:** 10.3389/fmicb.2022.1017278

**Published:** 2022-10-04

**Authors:** Giarlã Cunha da Silva, Ciro César Rossi, Jéssica Nogueira Rosa, Newton Moreno Sanches, Daniela Lopes Cardoso, Yanwen Li, Adam A. Witney, Kate A. Gould, Patrícia Pereira Fontes, Anastasia J. Callaghan, Janine Thérèse Bossé, Paul Richard Langford, Denise Mara Soares Bazzolli

**Affiliations:** ^1^Laboratório de Genética Molecular de Bactérias, Departamento de Microbiologia, Instituto de Biotecnologia Aplicada à Agropecuária—Bioagro, Universidade Federal de Viçosa, Viçosa, Brazil; ^2^Departamento de Bioquímica e Biologia Molecular, Universidade Federal de Viçosa, Viçosa, Brazil; ^3^School of Biological Sciences and Institute of Biological and Biomedical Sciences, University of Portsmouth, Portsmouth, United Kingdom; ^4^Section of Pediatric Infectious Disease, Department of Infectious Disease, Imperial College London, London, United Kingdom; ^5^Institute for Infection and Immunity, St. George’s, University of London, London, United Kingdom; ^6^Departamento de Microbiologia, Universidade Federal de Viçosa, Viçosa, Brazil

**Keywords:** *Pasteurellaceae*, porcine pleuropneumonia, *trans*-acting small RNA, extracellular vesicles, *Galleria mellonella*

## Abstract

The RNA chaperone Hfq promotes the association of small RNAs (sRNAs) with cognate mRNAs, controlling the expression of bacterial phenotype. *Actinobacillus pleuropneumoniae hfq* mutants strains are attenuated for virulence in pigs, impaired in the ability to form biofilms, and more susceptible to stress, but knowledge of the extent of sRNA involvement is limited. Here, using *A. pleuropneumoniae* strain MIDG2331 (serovar 8), 14 sRNAs were identified by co-immunoprecipitation with Hfq and the expression of eight, identified as *trans*-acting sRNAs, were confirmed by Northern blotting. We focused on one of these sRNAs, named Rna01, containing a putative promoter for RpoE (stress regulon) recognition. Knockout mutants of *rna01* and a double knockout mutant of *rna01* and *hfq,* both had decreased biofilm formation and hemolytic activity, attenuation for virulence in *Galleria mellonella,* altered stress susceptibility, and an altered outer membrane protein profile. Rna01 affected extracellular vesicle production, size and toxicity in *G. mellonella*. qRT-PCR analysis of *rna01* and putative cognate mRNA targets indicated that Rna01 is associated with the extracytoplasmic stress response. This work increases our understanding of the multilayered and complex nature of the influence of Hfq-dependent sRNAs on the physiology and virulence of *A. pleuropneumoniae*.

## Introduction

*Actinobacillus pleuropneumoniae* is the etiological agent of porcine pleuropneumonia, a contagious and severe respiratory disease with high lethality, economic impact, and worldwide distribution (Pattison et al., 1957; Sassu et al., 2018). Currently, 19 serovars of *A. pleuropneumoniae* are known (Stringer et al., 2021). Although virulence of *A. pleuropneumoniae* is multifactorial and complex (Pereira et al., 2018), it is strongly related to the production of different combinations of the pore-forming and/or cytolytic repeat-in-toxin (RTX) proteins ApxI, ApxII, and ApxIII (Frey, 2011). We and other researchers have shown that the RNA chaperone Hfq influences, in a serovar-dependent manner, different phenotypes of *A. pleuropneumoniae*, including adherence, susceptibility to stress conditions, and virulence (Subashchandrabose et al., 2013; Pereira et al., 2015; Crispim et al., 2020).

The Hfq protein forms a homo-hexameric ring and it is involved in several essential processes, such as rRNA processing, ribosome biogenesis, tRNA maturation, control of mRNA translation, DNA compaction, and activity of c-di-GMP metabolic enzymes (Kavita et al., 2018; dos Santos et al., 2019; Fu et al., 2021). However, its most prominent activity involves dynamically binding to small non-coding RNA molecules (sRNAs; Quendera et al., 2020). Hfq typically interacts with sRNAs with its proximal pore, *via* their Rho-independent terminators ending with 4–6 uridines, while the distal face of Hfq recognizes A-rich motifs that are often present in mRNA targets (Otaka et al., 2011; Sauer and Weichenrieder, 2011). This initial interaction then prompts Hfq to sweep the sRNA from the core to establish the sRNA-mRNA interaction (Santiago-Frangos and Woodson, 2018). Hfq acts thus as an RNA “matchmaker” that binds to several sRNAs and ensures their binding to equally diverse mRNA targets, leading to pleiotropic effects that make it a global regulator of post-transcriptional gene expression in bacteria (Updegrove et al., 2016).

Bacterial sRNAs are a diverse class of molecules, ranging from 50 to 500 nucleotides in length, that may act in *cis* (anti-sense RNAs) or in *trans*. *Trans*-acting sRNAs are not in the same locus of the target, and present partial complementarity to their mRNA cognates. They act through either blocking the ribosome binding site (RBS) or preventing translation initiation, or by destabilizing RBS sequestering RNA structures, favoring translation. Hfq interaction with RNAs can lead to degradation by RNase E or by polyadenylation of the 3′ UTR region (Vogel and Luisi, 2011; Carrier et al., 2018). The partial complementarity between the sRNA-mRNA pair and the small span of the pairing sites (about 7–12 nucleotides) allows a single sRNA to bind a variety of targets, forming a complex and intricate network of gene regulation (Nitzan et al., 2017; Carrier et al., 2018).

Previously, our group has combined computational prediction and experimental methods to show that the genome of *A. pleuropneumoniae* 5b L20 encodes several sRNAs, including many sRNAs not yet characterized (Rossi et al., 2016). Although not all sRNAs depend on the activity of Hfq, current data indicate that elements of the stress response and virulence of *A. pleuropneumoniae* are controlled by Hfq-dependent sRNAs (Subashchandrabose et al., 2013; Pereira et al., 2015; Crispim et al., 2020), but their identity is largely unknown. Thus, the goals of this study were to identify sRNAs that act through binding Hfq, and determine their role in the fitness and pathogenicity in *A. pleuropneumoniae*.

## Materials and methods

### *Actinobacillus pleuropneumoniae* strains, growth, and maintenance conditions

All experiments for the identification of Hfq-dependent sRNAs were conducted with strains described in Table 1. The two *A. pleuropneumoniae* WT strains used were serovar 5 L20 (Foote et al., 2008), and serovar 8 MIDG2331 (Bossé et al., 2016) hereafter referred to as Ap8WT which was used to derive mutants in genes of interest. Strains were cultivated aerobically in brain heart infusion (BHI, BD—237,500) broth supplemented with nicotinamide adenine dinucleotide (NAD, 10 μg. ml^−1^, Sigma-Aldrich—N0632-5G) at 37°C in an orbital incubator (180 rpm). For anaerobic growth, BHI broth was prepared with removal of oxygen and addition of N_2,_ according to Uchino and Ken-Ichiro (2011), with strains being cultivated in hermetically sealed bottles without shaking at 37°C until they reached early stationary phase, OD_600_: ~2.5 and 2.36 × 10^13^ CFU/ml for aerobiosis and DO_600_ = ~1 and 2.64 × 10^10^ CFU/ml for anaerobiosis.

**Table 1:** *Actinobacillus pleuropneumoniae* strains and mutants used in this study.

**Strain**	**Description**	**Reference**
**Serotype 5**		
Serotype 5b L20	Reference strain of serotype 5b/ used as positive control in biofilm assay	Foote et al., 2008
**Serotype 8**		
MIDG2331(Ap8WT)	Wild type strain from UK (clinical isolate)	Bossé et al., 2016
Ap8*hfq*::3XFLAG	MIDG2331 strain containing the *hfq* gene tagged with 3XFLAG tag at the C-terminus	Crispim et al., 2020
Ap8∆*hfq*	MIDG2331 ∆*hfq* mutant strain	Crispim et al., 2020
Ap8∆*rna*01	MIDG2331 ∆*rna*01 mutant	This study
Ap8∆*hfq*∆*rna*01	MIDG2331 ∆*hfq*∆*rna*01 mutant	This study

### Hfq co-immunoprecipitation and RNA sequencing

Cells from Ap8*hfq*::3xFLAG strain were cultured in 100 ml BHI broth until early stationary phase, split into aliquots of 50 ml (Test, samples from Ap8WT, Ap8*hfq*::3xFLAG and Ap8∆*hfq* subjected to co-IP; and Control, samples from same strains not subjected to co-IP), and centrifuged for 20 min at 5,000 *g* at 4°C. The supernatant was discarded, and pellets were washed twice in 1.0 ml nuclease/protease free PBS (50TAB, Sigma-Aldrich, United States), and transferred into 2.0 ml microtubes. The microtubes were recentrifuged and pellets frozen in liquid nitrogen and kept at −80°C until use, when they were thawed on ice and re-resuspended in 1.0 ml ice cold lysis buffer (20 mM Tris pH 8, 150 mM KCl, 1 mM MgCl_2_, and 1 mM DTT) before being transferred to tubes containing Lysing Matrix B beads (MP Biomedicals, CA, United States). Lysis was conducted in the FastPrep-24™apparatus (MP Biomedicals, CA, United States), setting program 5. This procedure was performed twice, with a 1 min interval of cooling of samples on ice between homogenizations. The lysate was cleared by centrifugation (40 min, 16,000 × *g* at 4°C), supernatants (800 μl) transferred to new tubes, to which 400 U.ml^−1^ of RNAsin inhibitor (Promega, WI, United States) had been added. Co-IP was performed as previously described (Sittka et al., 2008) with modifications. Briefly, Hfq-3xFLAG expressed by Ap8 *hfq*::3xFLAG was co-immunoprecipitated with associated RNAs by the addition of 35 μl anti-FLAG M2 monoclonal antibody (F1804—Sigma-Aldrich, MO, United States), following the manufacturer’s instructions. The tubes were gently agitated for 60 min at 4°C, and 75 μl of prewashed Protein A Sepharose (P6649—Sigma-Aldrich) added, and gently agitated for an additional 60 min. After the agitation period, the tubes were centrifuged (1,000 *g* 1 min at 4°C), the supernatant was discarded, and the beads washed with lysis buffer as described above. TRIzol (1 ml, Invitrogen, CA, United States) was added to the beads, and RNA isolation was performed as suggested by the manufacturers. The co-IP procedure was also performed with Ap8WT and Ap8∆*hfq* strains as controls to assess the effectiveness of the technique to enrich for Hfq-associated RNAs. Total RNA was obtained and analyzed using a Bioanalyzer (Agilent Technologies, CA, United States). RNA sequencing was done using Ion Total RNA-seq kit v2 (Life Technologies), according to the manufacturers’ protocols. Samples were loaded onto a 318 chip and sequenced on Ion torrent-PGM (Life Technologies) using default manufacturer’s parameters (single-end, forward sequencing).

### Mapping, assembly, and analysis of RNA sequencing results

RNA sequence reads were mapped onto the genome of *A. pleuropneumoniae* MIDG2331 (Ap8WT strain; Genbank access LN908249) using Burrows-Wheeler Aligner (BWA-MEM algorithm, default parameters) version 0.7.10 (Li and Durbin, 2010). The resulting bam files were uploaded to NCBI’s Short Read Archive (SRA, experiment SRX810211). Transcriptome assembly was made with Cufflinks version 2.2.1 (Ghosh and Chan, 2016). Results were analyzed using the sequence viewers JBrowse and Tablet (Milne et al., 2010; Buels et al., 2016). Putative *trans* sRNA candidates were identified by increased reads within intergenic regions in the annotated genome. After delimiting the sRNA candidates, the effectiveness of co-IP was evaluated by normalizing read counts as reads per kilobase million (RPKM). Normalized reads of the sRNA candidates from the three strains in aerobic and anaerobic conditions were used to plot a heatmap using the ggplot R package. Read coverage of the candidates was visualized using the integrative genome viewer (Robinson et al., 2020). The sRNA candidates were evaluated regarding their novelty by searching in the Rfam database.

### Northern blotting

The Ap8WT, Ap8∆*hfq*, and Ap8*hfq*::3XFLAG strains were cultivated until early stationary phase under aerobic and anaerobic conditions as described above. Bacterial pellets from 1 ml of culture were disrupted with Lysing Matrix B tubes (MP Biomedicals), and RNA extraction was then performed with the miRNeasy Mini Kit (Qiagen) following the manufacturer’s instructions. The resulting total RNA was quantified and treated with one unit of RQ1 DNase (Promega) per μg of nucleic acid and incubated for 60 min at 37°C. DNA removal from the sample was confirmed by PCR with the oligonucleotide pair APP5SF and APP5SR for the 5S rRNA (Table 2) as previously described (Rossi et al., 2016). Total treated RNA (10 μg) was loaded and run on a 10% TBE-urea polyacrylamide gel and transferred by electrophoresis to a Brightstar Plus nylon membrane (Applied Biosystems, CA, United States). Hybridization was conducted with the DIG High Prime DNA Labeling and Detection Starter kit II (Roche, Switzerland), according to the manufacturer’s instructions. Primers designed for each sRNA (Table 2) were used to construct 109 ± 18 bp digoxigenin-marked probes with the PCR DIG probe synthesis kit (Roche). As hybridization controls, all membranes were re-hybridized with probes for the rRNA 5S.

**Table 2:** Primers used in this work.

**Primer**	**Sequence 5′-3′**	**Amplicon size (bp)**	**Purpose**
Rna01F	CCGGCACCAAGAAAGCGAT	100	Synthesis of probes for Northern blotting
Rna01R	AAACGGCTCAGTCTTAAATAACGC		
Rna02F	GTTCACATTGTAAGAAGAAGAAGCA	159	
Rna02R	CCTCAACTTAGGGGCTACTCG		
Rna03F	CTTACCAGTCAGAGTATCATTGG	96	
Rna03R	TGCGGCACTACTTTTAAGAAGCC		
Rna04F	TTACAATGTGGTCGTTCTATGACAA	101	
Rna04R	TCCTAGCCAATATATTAGGAATGAAT		
Rna05F	TGTTGTGTTTGCATATTGGTCTAGG	104	
Rna05R	TGGACGGTTATAAACCAAAAAGGT		
Rna08F	ACGACTATCTCTTCGACTGCT	103	
Rna08R	GCATCAATGTGCGGGCAAAG		
Rna09F	GCTGAACCGACAGCGGAA	103	
Rna09R	TCCTTAGGTAAGGCGAGCTTC		
Rna10F	ATCGGCGATTTAATATTCGGGC	106	
Rna10R	GCAAGCCAACTCGTATAGGG		
Rna12F	AGCGATTGTTATCCGGTCGT	147	
Rna12R	AGACGGTCAGAAGCTCCTTT		
Rna13F	CACTAAGGTTGGGGCAAAGG	102	
Rna13R	GCAATAAAATAATACGACCG		
Rna14F	ACATTAAGCACATCTAAGAG	100	
Rna14R	AGCAAGTAGAAGGAGTTCTA		
APP5SF	GCGATGCCCTACTCTCACAT	100	Positive control for Northern blotting and RT-PCR
APP5SR	GAGTGCTGTGGCTCTACCTG		
Rna01_up	TGCCGTTAGCTTAGTGAGATTC	683	Confirmation of replacement of the *rna01* gene by recombination using natural transformation
dfrA_5’out	CACGGTTCTCATCCTAATTCCTCC		
sbcB_for	ACGATGAAATGACCCGTTATACC	453	Analysis of the flanking genes to confirm no polar effect in ∆*rna01* mutant strain
sbcB_rev	CTTGATTTTGATTAGTTGGGTGCC		
eriC_for	AAGTGCAAACGAGCTTATGGC	265	
eriC_rev	GCACACCACACCGCATAATG		
ompp2B_F	CGTAACCACCCTCAGCAT	73	Used in qPCR analysis
ompp2B_R	GCATATGGTTTAGGTGCGGT		
ata2_F	GGTTTCCAATCCATCGCTCG	118	
ata2_R	CAGAACCGACACCCATAGC		
Rna01_F1	CTAACTGACAGAATTTATGTAAG	72	
Rna01_F2	ACCAAGAAAGCGATGCCG		
tolR_F	AATACACTCTTCCTTGTCGCTGC	100	
tolR_R	ACCCTGCGTTCTTAATACCCG		
gyrA_F	GTCGTGGCGGTAAAGGTAAA	111	Endogenous control for qPCR analysis
gyrA_R	GACCACGGCTTGAGAAACAT		

### *In silico* analysis of Rna01

From the RNAseq/Northern blotting and initial *in silico* results, we decided to focus on one of the sRNA candidates discovered, named Rna01. The promoter sequence of Rna01 was predicted with Softberry BProm (available at http://www.softberry.com), and the putative Hfq-binding sequence (Holmqvist et al., 2016) was inspected visually. Additionally, we searched for homologs by alignment of the Ap8WT *rna01* sequence against NCBI’s Genbank and PATRIC databases, using BLASTn with a 70% cut-off for both coverage and sequence identity. The alignment of the homolog sequences was done using Clustal Omega (Sievers and Higgins, 2014). The alignments of the putative promoter and Hfq binding sequences were done using WebLogo (Crooks et al., 2004).

### Analysis of mRNA targets

Potential mRNA targets of Rna01 were searched in the genome of Ap8WT by combining TargetRNA2 and CopraRNA prediction tools (Kery et al., 2014; Wright et al., 2014), considering a value of *p* below 0.05 for predictions in both tools and a false discovery rate (fdr) below 0.5 (Benjamini and Hochberg, 1995) for CopraRNA. The MIDG2331 genome was used as a reference for target prediction in TargetRNA2, while CopraRNA required three Rna01 homologs, which were extracted from GenBank using NCBI accession numbers NZ_LN908249 for *A. pleuropneumoniae* serovar 8 MIDG2331, NC_009053 for *A. pleuropneumoniae* serovar 5 str. L20 and NC_010942 for *A. pleuropneumoniae* serovar 7 str. AP76. We also did manual inspections searching for potential targets by aligning the predicted seed regions with the genome of MIDG2331. Target’s functions were investigated using the UniProT database as reference (The UniProT Consortium, 2019). Conservation of putative seed regions of Rna01 among the homologs was verified using Jalview (Waterhouse et al., 2009) using the homologs alignment. To investigate protein–protein interaction among the targets, the predicted mRNA targets were analyzed in STRING (Szklarczyk et al., 2019) using a moderate confidence 0.400 and Markov clustering method (MCL) with inflation parameter 1.1.

### Comparative analysis of Rna01 and extracytoplasmic stress associated sRNAs

To investigate Rna01 as a possibly novel sRNA associated with extracytoplasmic stress responses, its homology with stress-associated sRNAs from other bacteria, such as MicA, MicF, MicC, MicL, OmrA, OmrB, RseX (all from *E. coli*), VrrA (*V. cholerae*), and RybB (*S. enterica*), was analyzed for sequence alignment with Clustal Omega (Sievers and Higgins, 2014). The identity matrix generated was plotted in a heatmap using the ggplot R package. Additionally, sRNAs structures and putative seed regions of OM targets were predicted using RNAfold and TargetRNA2, respectively.

### Construction of Rna01 knockout mutants

Two different Rna01 knockout strains were constructed in this work: a single *rna01* mutant (Ap8∆*rna01*) from the parental *A. pleuropneumoniae* Ap8WT strain and a double *rna01* and *hfq* mutant (Ap8∆*hfq*∆*rna01*) from the previous Ap8∆*hfq* strain (Table 1). A DNA cassette was designed to allow the replacement of the *rna01* gene by natural transformation, using the method previously described (Bossé et al., 2014) with modifications. This construct contained 500 bp of the upstream and downstream regions of *rna01* (flanking sequences), with the *rna01* gene being interrupted by the trimethoprim resistance gene *dfrA14* from the plasmid pM3389T (Bossé et al., 2015), which was put under the control of the *sodC* promoter and was immediately followed downstream by the 9 bp 5′ ACAAGCGGT3′ DNA Uptake Sequence (DUS), which enables efficient natural transformation by *A. pleuropneumoniae* (Redfield et al., 2006), and a Rho-independent terminator sequence (5′ AGCCGCCTAATGAGCGGGCTTTTTTTT3′), as described in Supplementary Figure 1. The entire 1,625 bp cassette was synthesized and cloned into the pEX4K vector by Eurofins Genomics (Germany). The plasmid was cloned into ultracompetent *E. coli* DH5α (Sambrook and Russell, 2006), purified with the Qiaprep Spin Miniprep kit (Qiagen), and linearized by digestion with NotI (Promega) at 37°C for 3 h, followed by heat inactivation at 65°C for 20 min. The linearized plasmid (1 μg DNA) was then used to naturally transform *A. pleuropneumoniae* strains as previously described (Bossé et al., 2014). Transformants were selected on BHI agar supplemented with 10 μg/ml NAD and 10 μg/ml trimethoprim. Correct replacement of the *rna01* gene with the *dfrA14* cassette was confirmed by PCR amplification of a 683 bp fragment using the primers RNA01_up (which binds further upstream than the sequence contained in the replacement construct, so that no residual donor DNA could be detected) and dfrA_5′out (Table 2), and sequencing. Absence of polar effects on the expression of the *rna01* flanking genes, *sbcB* and *eriC*, were confirmed by RT-PCR. Briefly, DNase-treated RNA, prepared as described above, was used to synthesize cDNA with the ImProm-II Reverse Transcription System kit (Promega), following the manufacturer’s suggestions. Subsequently, a PCR was performed with the primers sbcB_for and sbcB_rev to detect the expression of the *scbB* gene, and eriC_for eriC_rev for the *eriC* gene (Table 2).

### *rna01* and targets expression by quantitative PCR

A total of 2 μg of RQ1 DNase-treated RNA was used to synthesize cDNA with the High-Capacity cDNA Reverse Transcription kit (Applied Biosystems). The expressions of the targets *ompP2B*, *tolR*, *ata_2*, and the *rna01* gene were evaluated by qRT-PCR in the Ap8WT, Ap8∆*rna01*, Ap8∆*hfq*, and Ap8∆*hfq*∆*rna01* strains (except for the ∆*rna01* strains to evaluate *rna01* expression). Reactions were performed in the CFX96 Touch Real-Time PCR Detection System (Bio-Rad) with primers depicted in Table 2. The R2 was calculated for each primer set, and the reaction efficiency was calculated. Amplifications were performed under the following conditions: 2 min at 95°C, followed by 40 cycles of 15 s at 95°C and 1 min at 60°C. After the amplification step, a melting curve was performed for each primer set. The relative quantification was done by a standard curve obtained for each gene, by the equation of the line relating average Ct and log10 of control cDNA concentration. The results were normalized with *gyrA* as reference gene.

### Phenotypic analysis of *rna01* mutants

#### Growth parameters

Aliquots from overnight cultures of Ap8WT, Ap8∆*hfq*, Ap8∆*rna*01, and Ap8∆*hfq*∆*rna*01 strains were transferred to a fresh 50 ml BHI-NAD broth with an initial starting OD_600_ = 0.1. Strains were cultivated under constant agitation (180 rpm) at 37°C in aerobic conditions, and ODs measured every 20 min for the first 5 h and, subsequently, every hour, for the next 12 h. The experiment was performed in biological triplicate.

#### Biofilm formation assay

Cell adherence was evaluated in microtiter plates as previously described (Stepanović et al., 2007), with modifications. Briefly, the Ap8WT, Ap8∆*hfq*, Ap8∆*rna*01, and Ap8∆*hfq*∆*rna*01 strains were grown overnight in BHI-NAD at 37°C and 5% CO_2_, the cultures adjusted to OD_600_ = 0.1, and 150 μl were transferred to the microtiter plate wells, followed by incubation at 37°C and 5% CO_2_ for 24 h. After this period, the wells were gently washed 3x with distilled water, stained with 150 μl of 0.1% crystal violet solution for 10 min, washed again with distilled water, then stained cells were resuspended in 95% ethanol, and kept at room temperature for 45 min. The OD_590_ was measured in a Multiskan Go spectrophotometer (Thermo Scientific). The experiment was performed in triplicate and negative controls consisted of wells to which sterile BHI-NAD was added at the beginning of the experiment. The *A. pleuropneumoniae* strain L20 was used as a positive control for biofilm formation (Foote et al., 2008).

#### Stress conditions susceptibility assay

The following agents and their concentrations were used in BHI-NAD agar to investigate the sensitivity of the Ap8WT, Ap8∆*hfq*, Ap8∆*rna*01, and Ap8∆*hfq*∆*rna*01 strains to osmotic stress (0.1 M KCl), oxidative stress (0.2 mM H_2_O_2_), temperature stress (42°C), and exposure to the antibiotics tylosin and ampicillin and measuring the minimum inhibitory concentration (MIC). Bacterial cultures with an initial OD_600_ of 1.0 were serially diluted in PBS to 10^−7^ and 10 μl of each 10-fold dilution was spotted onto each selective stress agar. As a control, cultures were plated on BHI-NAD agar containing no stressing agent. All plates were incubated at 37°C for 24 h. Antimicrobial sensitivity was evaluated by the microdilution method, according to the Clinical and Laboratory Standards Institute recommendations—Document M31-A3 (CLSI, 2008). After incubating the microplates at 37°C for 24 h, 50 μl of resazurin 0.01% (Sigma-Aldrich—R2127) were added to each well and the microplate was incubated again for 1 h at 37°C, in the dark. The MIC of each antibiotic was considered as the lowest concentration at which resazurin was not reduced, i.e., no change in color from blue to pink. BHI-NAD broth was used without the addition of antibiotics as a positive control for bacterial growth.

#### Virulence assay in *Galleria mellonella*

Virulence effects of the deletion of the *rna01* gene, alone or with *hfq*, were evaluated using *G. mellonella* as an infection model. Propagation and rearing of *G. mellonella* larvae and experiments with last-instar larvae were conducted as previously described (Pereira et al., 2015). Survival was monitored every 24 h, for 96 h post-infection. Negative controls consisted of larvae inoculated with PBS. Tests were performed in biological and experimental triplicate, with 10 larvae per replicate. Melanin production was quantified as previously described (Jorjão et al., 2018), with modifications. Briefly, incisions were made in the larvae’s prolegs with a micro scissor to allow the hemolymph to leak. The haemolymph was collected with a micropipette and transferred to refrigerated microtubes, which were centrifuged at 9,500 *g* for 10 min at 4°C. The supernatant was diluted in anticoagulant solution (Mead et al., 1986), and the OD_405_ measured.

#### Hemolytic activity

The hemolytic activity of the Ap8WT, Ap8∆*hfq*, Ap8∆*rna*01, and Ap8∆*hfq*∆*rna*01 strains in defibrinated sheep blood (Ebefarma, RJ, Brazil) was evaluated as previously described (Shin et al., 1999), with modifications. Cultures were initially adjusted to OD_600_ = 0.1 and cultivated in 50 ml BHI-NAD under agitation (180 rpm) at 37°C in aerobic conditions until late exponential phase (OD_600_ ~ 2.5). Subsequently, 5 ml of cultures were centrifuged (5,000 *g* for 5 min), and re-suspended in PBS to OD_600_ = 2.0. An aliquot of this suspension (0.75 ml) was added to an equal volume of 1% sheep blood and incubated for 1 h at room temperature (25°C) under gentle agitation. Then, samples were centrifuged (1,000 × *g*/5 min), and the OD_405_ measured. Negative and positive controls consisted of blood suspensions in PBS alone or with 1% Tween 20, respectively. Percentage of hemolysis was calculated using the following formula: hemolysis (%) = [(sample OD_405_ – PBS OD_405_)/(Tween20 OD_405_ − PBS OD_405_) × 100]. The experiment was performed in triplicate.

### Outer membrane protein extraction

The fractions of outer membrane proteins (OMPs) from the Ap8WT, Ap8∆*hfq*, Ap8∆*rna*01, and Ap8∆*hfq*∆*rna*01 strains were obtained following the “method 1” previously described (Thein et al., 2010), with 25 ml of cells culture being cultivated in BHI-NAD broth at 37°C and 180 rpm until exponential and stationary phases (~6 and ~ 14 h, respectively). The proteins were separated by 12% SDS-PAGE followed by Coomassie blue staining.

### Extracellular vesicle extraction and analysis

#### Hydrostatic filtration to obtain EVs

Extracellular vesicles (EVs) were obtained from the Ap8WT, Ap8∆*hfq*, Ap8∆*rna*01, and Ap8∆*hfq*∆*rna*01 strains using the hydrostatic filtration method for purification (Antenucci et al., 2017). Aliquots from overnight cultures of the strains were inoculated into 600 ml of fresh BHI such that the initial starting OD_600_ = 0.01, and cultivated until late exponential phase (OD_600_ ~ 2.5). After purification, the volume of EVs recovered from each strain was equally adjusted to enable comparison of EVs production among the strains by relative protein abundance. Relative quantification was done by use of a standard curve obtained using bovine serum albumin (BSA—Sigma-Aldrich–A-4503) and the Bradford reagent (Sigma-Aldrich B6916).

#### Transmission electron microscopy of EVs

For transmission electron microscopy, 10 μl containing ~0.3 μg of EV samples from each strain above were placed on a formvar coated grid and stained with 3% uranyl acetate. The visualization was performed in a Zeiss EM 109 Electron Transmission Microscope in the Facility Center of Microscopy and Microanalysis at the Universidade Federal de Viçosa.

#### EV size measurement

The size of the EVs obtained was measured in an electrophoretic light scattering apparatus (Zetasizer Nano ZS, Malvern Instruments, United Kingdom). The data were analyzed with Malvern zetasizer software version 7.11 to obtain the average hydrodynamic diameter of the particles in solution. The measurements were conducted at 25°C with three replica runs of 5 min each, and the average intensity weighted diameter was calculated. To measure these parameters, 30 μg of EVs diluted with PBS pH 7, with a refractive index of 1.332 and a viscosity of 0.9043, were used.

#### Protein’s profile of EVs

To analyze the protein profile, EVs were dissolved in lysis buffer (50 mM Tris-Cl pH 6.8; 100 mM dithiothreitol; 2% SDS; 0.1% bromophenol blue; 10% glycerol), and heated for 10 min at 100°C. The samples were separated in a 12% SDS-PAGE gel, and stained with Coomassie blue (Green and Sambrook, 2012).

#### Toxicity of EVs produced by *Actinobacillus pleuropneumoniae* for *Galleria mellonella*

The toxicity of EVs produced by the strains was evaluated as described above by inoculating larvae of *G. mellonella* with 20 μg of EVs from each strain. Negative controls consisted of larvae inoculated with PBS. Tests were performed in biological and experimental triplicate, with 10 larvae per replicate. Survival was monitored every 24 h, for 96 h post-infection. Survival curves were plotted as described above. The melanization levels of the larvae infected with EVs were evaluated by infecting larvae with 3 μg of BEVs from each strain, as described above.

### Statistical analysis

For toxicity in the *G. mellonella* assay, survival curves were plotted using the Kaplan–Meier method (Kleinbaum and Klein, 2012), and differences in survival were estimated by using the log rank test using the software *R*, version 2.13.0, with values of *p* < 0.05 considered as statistically significant. For melanization and phenotypic analysis, the differences were analyzed using analysis of variance (ANOVA) followed by the Tuckey test for multiple comparisons with *p* values <0.05 considered as statistically significant. For the quantitative expression of genes, the t-test was used considering *p* values <0.1 as statistically significant.

## Results

### The Hfq-FLAG-tagged sample was enriched with sRNAs

**Figure 1:**
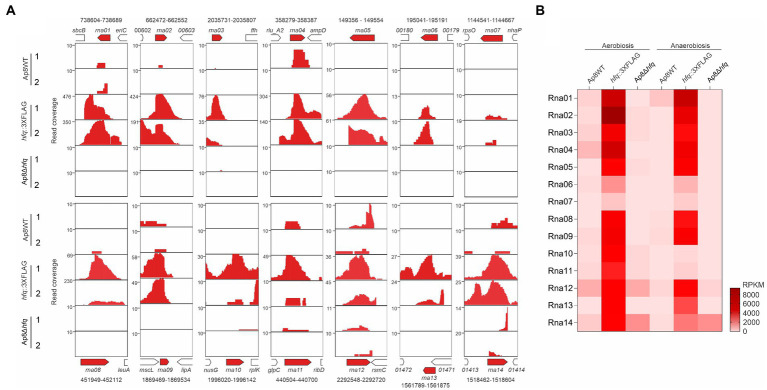
Identification of novel sRNA candidates associated with the chaperone Hfq. **(A)** Mapping regions of the RNA sequences from the WT, Ap8*hfq*::3XFLAG, and Ap8Δ*hfq* strains showing increased intergenic reads in aerobiosis (1) and anaerobiosis (2), corresponding to the 14 sRNA candidates selected in this study. Genomic coordinates of the sRNAs are represented above (*rna01* to *rna07*) or below (*rna08* to *rna14*) of the open reading frames. **(B)** Heatmap showing the coverage, in reads per kilobase of transcript, per million mapped reads (RPKM), of the 14 putative novel sRNAs identified by co-IP.

Co-immunoprecipitation (Co-IP) assays were performed to obtain sRNAs associated with the Hfq chaperone. Co-IP assays resulted in 31.5, 135.5, and 18.1 ng of RNA total/μl being isolated from Ap8WT (MIDG2331 wild-type), Ap8*hfq*::3XFLAG (MIDG2331 containing an *hfq* gene with 3XFLAG tag at the C-terminus), and Ap8Δ*hfq* (MIDG2331 *hfq* mutant), respectively, showing the highest enrichment for the *hfq*::3XFLAG strain (4.3x Ap8WT and 7.5x compare to the Ap8Δ*hfq* strains). Sequencing of co-immunoprecipitated RNAs produced an average of 924,834 ± 66,055 reads for each strain. To improve sRNA discovery, we merged the data from aero/anaerobiosis, which were then mapped to the genome of MIDG2331, available at the NCBI’s Sequence Read Archive (SRA, access SRX810211). The search for abundant intergenic transcripts led to the discovery of 14 sRNA candidates, named here Rna01-14 (Table 3; Figure 1A). Normalized reads showed higher values for the co-immunoprecipitated sRNA candidates in the RNAseq data of Ap8*hfq*::3XFLAG in comparison to WT and Ap8∆*hfq* strains, showing the efficacy of the technique (Figure 1B). From the analysis of the sequences in the Rfam database, Rna06, Rna07 and Rna11 were not investigated further, as they were not *trans*-acting, corresponding to the RNAs TPP riboswitch, ribosomal S15 leader, and the FMN riboswitch, respectively.

**Table 3:** Regulatory sRNAs identified in this work by Hfq Co-immunoprecipitation in *Actinobacillus pleuropneumoniae.*

**sRNA**	**Genome position** ^**a**^	**Strand**	**Size (bp)**	***Upstream* gene**	***Downstream* gene**	**Rfam classification (Access)**
Rna01	738,604–738,689	−	86	*eriC*	*sbcB*	No match
Rna02	662,472–662,552	+	81	MIDG2331_00602	MIDG2331_00603	AaHKsRNA020 (RF02898)
Rna03	2,035,731–2,035,807	+	77	tRNA-Asn(gtt)	*ffh*	No match
Rna04	358,279–358,387	+	109	*rlu_A2*	*ampD*	No match
Rna05	149,356–149,554	−	203	*gcvA*	MIDG2331_00135	GcvB RNA (RF00022)
Rna06	195,041–195,191	−	150	MIDG2331_00179	MIDG2331_00180	TPP riboswitch RNA (RF00059)
Rna07	1,144,541–1,144,667	+	127	*nhaP*	*rpsO*	Ribosomal S15 leader (RF00114)
Rna08	451,949–452,112	+	163	MIDG2331_00410	*leuA*	*Actinobacillus* sRNA 14 (RF02860)
Rna09	1,869,469–1,869,534	+	191	*mscL*	*lipA*	No match
Rna10	1,996,020–1,996,142	+	124	*nusG*	*rplK*	No match
Rna11	440,504–440,700	+	197	*glpC*	*ribD*	FMN riboswitch (RF00050)
Rna12	2,292,548–2,292,720	+	173	*comF*	*rsmC*	No match
Rna13	1,561,789–1,561,875	−	87	MIDG2331_01471	MIDG2331_01472	No match
Rna14	1,518,462–1,518,604	+	143	MIDG2331_01413	MIDG2331_01414	No match

a Position in the genome of *A. pleuropneumoniae* MIDG2331 strain (Genbank Access LN908249).

Eight candidates, Rna01, Rna03, Rna04, Rna09, Rna10, Rna12, Rna13, and Rna14 were considered as novel sRNA candidates since there was no previous identification/classification in Rfam database version 14.7, available at http://rfam.xfam.org/ (Kalvari et al., 2021). These were considered as possible *trans*-acting sRNAs for the further analysis. Two candidates, Rna13 and Rna14, were located in an integrative and conjugative element, ICE*Apl1*, so far only found in some serovar eight isolates of *A. pleuropneumoniae* (Bossé et al., 2016). Other *trans-acting s*RNAs have been described before. Rna02 is classified in the yet poorly characterized AaHKsRNA020 family (Rfam access RF02898). Rna05 is the well-studied sRNA GcvB, known to bind several sRNAs, mainly involved in amino acid biosynthesis and transport (Sharma et al., 2011; Gulliver et al., 2018; Lalaouna et al., 2019). This sRNA corresponds to the sRNA Arrc01, previously identified by our group by computational approaches (Rossi et al., 2016). The same applies to Rna08, which corresponds to sRNA Arrc14 (Rossi et al., 2016).

### Seven Hfq-bound sRNAs were confirmed by Northern blotting

**Figure 2:**
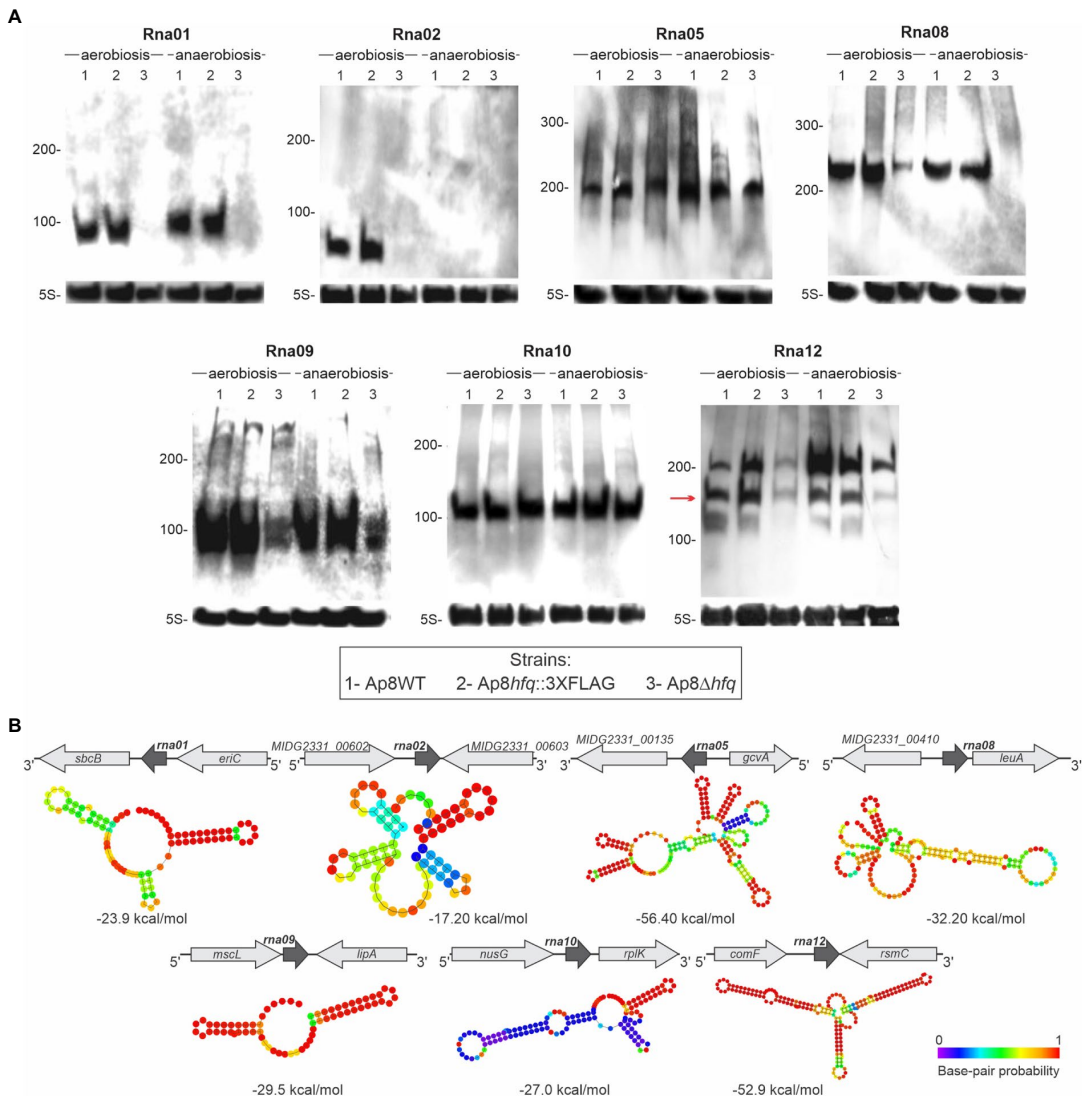
Validation of the expression of Hfq-associated sRNAs identified in this work. **(A)** Expression of sRNAs identified by co-IP using Hfq. Expression was assessed in *Actinobacillus pleuropneumoniae* Ap8WT (1), Ap8*hfq*::3XFLAG (2), and Ap8∆*hfq* (3) strains growing under aerobic and anaerobic conditions until late exponential phase. All membranes were reprobed using the rRNA 5S, expressed constitutively. The red arrow represents the band that corresponds to the predicted size for the respective sRNA. The experiment was performed in triplicate. **(B)** Genic organization and secondary structure of confirmed sRNA candidates, predicted by RNAfold (Lorenz et al., 2011). Free energy (ΔG) of sRNA structures is showed next to each structure.

The expression of seven of the 11 candidates obtained from co-IP was confirmed by Northern blotting in aerobic and anaerobic conditions from the total RNA of the Ap8WT, Ap8*hfq*::3XFLAG and Ap8Δ*hfq* strains (Figure 2A). The genomic organization and predicted secondary structures of the confirmed sRNAs are shown in Figure 2B. Most of the sRNAs show stable secondary structures, based on the base-pair probability and free-energy (ΔG). Northern blot results for four of the sRNAs analyzed, Rna01, Rna02, Rna08, and Rna12, suggest their instability in the absence of Hfq, as they were either undetected in the Ap8∆*hfq* mutant or were detected in lower concentrations than in the WT or Ap8*hfq*::3XFLAG strains. Rna02 was mostly (or exclusively) expressed during aerobic growth (Figure 2A).

The sRNAs named Rna03, Rna04, Rna13, and Rna14 were not abundantly expressed in the conditions evaluated, therefore they were not detected in the Northern blot. Although most RNA bands match their approximate expected size, Rna12 was detected with additional shorter bands, which could be by products of RNA processing or degradation products under the conditions analyzed. From the confirmed sRNAs, based on the expression of the Rna01 in aerobiosis and anaerobiosis conditions and the indicative dependence on the chaperone Hfq, both revealed in the Northern blot results, we selected this sRNA for further characterization.

### The *rna01* gene structure is conserved among *Pasteurellaceae*

**Figure 3:**
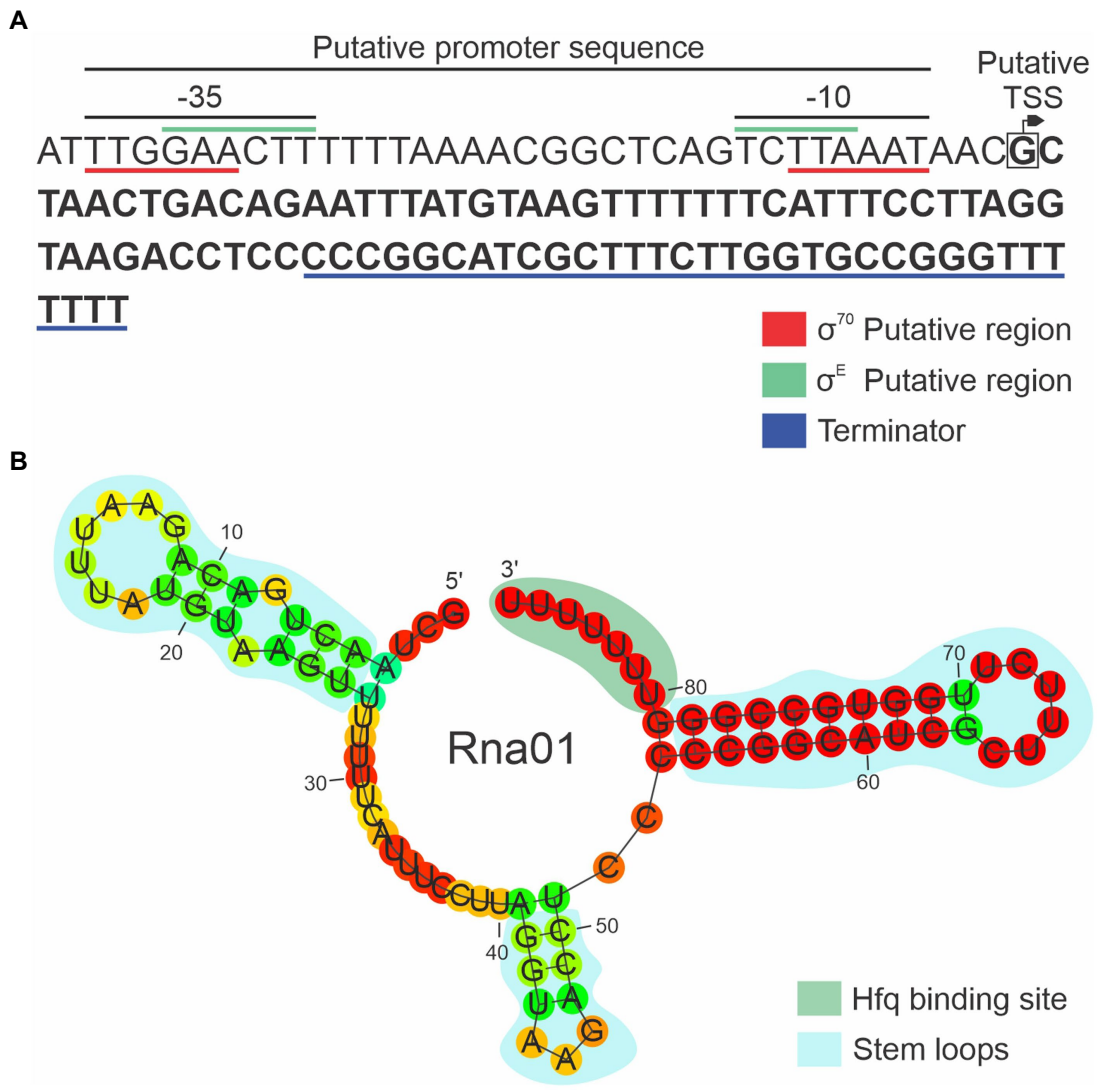
Characterization of the new sRNA Rna01. **(A)** Coding sequence of *rna01*. The putative promoter and Rho-independent terminator are shown. The putative transcriptional start site (TSS) is represented by the box with an arrow, and the −35 and −10 regions for each putative promoter sequences are underlined. The Rna01 sequence is highlighted in bold. **(B)** Secondary structure of Rna01 predicted using Rnafold software. The putative stem loops and putative Hfq binding site are shown.

The 86 bp *rna01* gene, encoding Rna01, is located in the intergenic region between *sbcB* and *eriC* on the opposite strand. Analysis of the sequence upstream of the predicted start site of *rna01* in *A. pleuropneumoniae* (Figure 3) revealed a putative RpoE/σ^E^ promoter sequence, GAACTT-16bp-TCTTA, with 6 bp between the end of the -10 and the start of the sRNA sequence. This promoter sequence matches the consensus for binding sites of the stress-response RpoE sigma factor in *A. pleuropneumoniae* (Bossé et al., 2010) and is similar to the RpoE consensus binding sites other bacteria such as *E. coli* (Raina et al., 1995) and *Salmonella* (Miticka et al., 2004). A sequence with similarity to the *E. coli* consensus binding site (TTGACA-17+/-2bp-TATAAT) for the housekeeping RpoD/σ^70^ polymerase (Shimada et al., 2014) was also seen upstream of *rna01* in MIDG2331, but the spacing is suboptimal (i.e., a sequence of TTGGAA-21bp-TTAAAT, with only 3 bp from the end of the -10 to the start of the sRNA). We also identified a putative Hfq-binding sequence (GGGUUUUUUU) which is part of the transcriptional terminator of Rna01 (Figure 3). The secondary structure of Rna01 had three predicted stem loops, a putative Rho-independent terminator, and a free energy (∆G) of -23.9 kcal/mol (Figure 3).

Rna01 homologs were found in a wide variety of members of the family *Pasteurellaceae* including: *Actinobacillus* spp., *Frederiksenia canicola*, *Glaesserella parasuis*, *Haemophilus ducreyi*, *Bibersteinia trehalosi*, *Mannheimia haemolytica*, and *Mannheimia varigena*. There were −35 and −10 sequences for both σ^70^ and σ^E^ promoters and Hfq-binding sites, with minor differences among homologs (Supplementary Figure 2).

### Rna01 potentially targets mRNAs encoding outer membrane proteins

**Figure 4:**
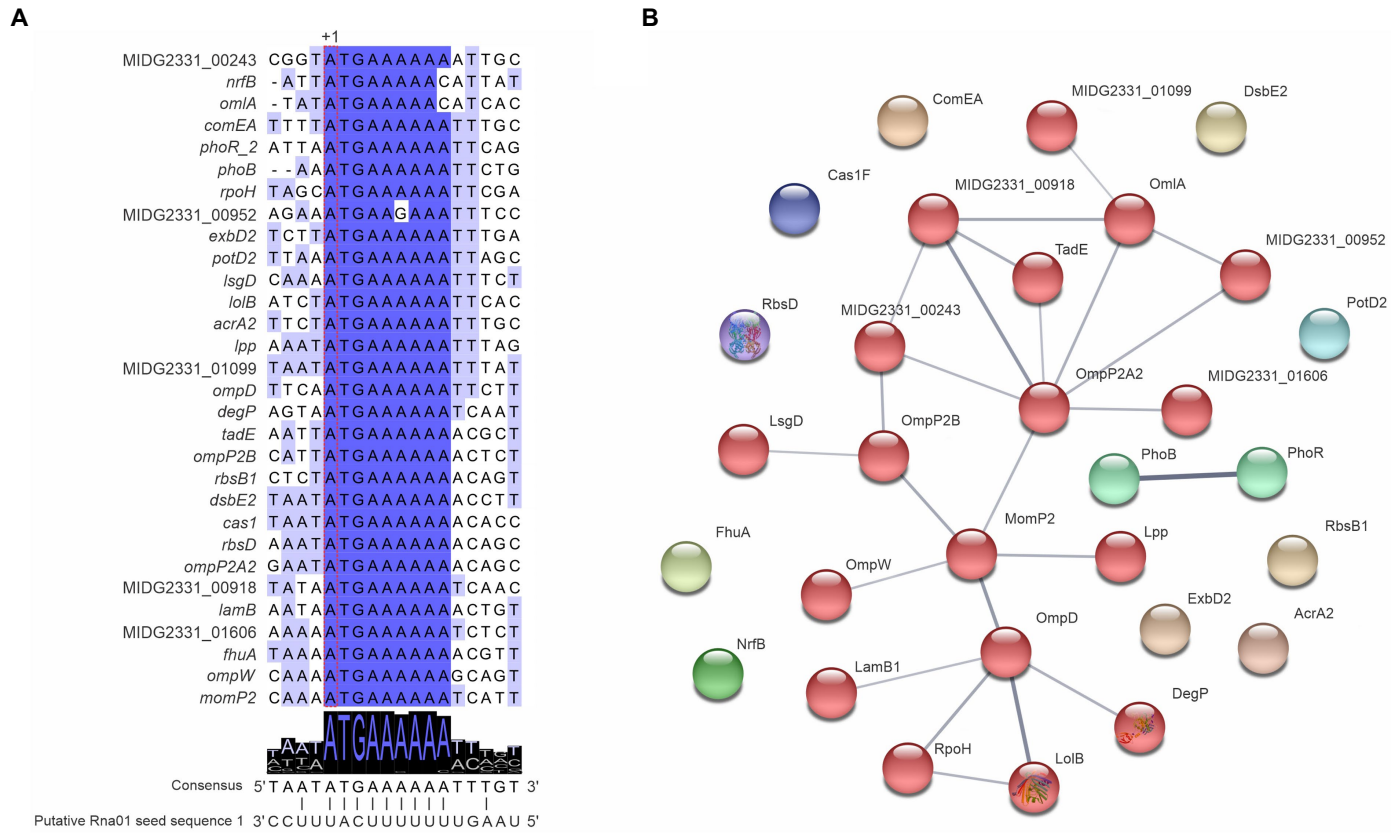
*Actinobacillus pleuropneumoniae* mRNA Targets predicted to be bound by Rna01. **(A)** Targets of Rna01 manually predicted in the MIDG2331 genome and their interaction with the seed region 1 of Rna01. Predicted translational start sites of Rna01 targets is represented by “+1.” **(B)** Interaction of manually predicted targets of Rna01. Grey lines represent protein–protein associations, and their thickness indicates the strength of data support. Associations are meant to be specific and meaningful, for example, proteins that jointly contribute to a shared function. Genes were analyzed with a moderate confidence 0.400 and Markov clustering method (MCL) with inflation parameter 1.1. Associated proteins share colors in the network.

The alignment of Rna01 homologs showed conserved sequences (Supplementary Figure 3). By manual prediction of targets based on the conserved sequence of Rna01, we found a substantial number of potential targets, including diverse genes involved with membrane processes (Figure 4A) with mostly high free energy (−4.92 to −12.55 kcal/mol; Supplementary Table 1). All the targets had a putative region of interaction around the ribosomal binding site (RBS) and translation start site (+1 translation), and were highly conserved (Figure 4A).

Rna01 targets predicted manually were compared against STRING databases using *A. pleuropneumoniae* serovar 5 L20 genome for reference, to enquire whether their products shared metabolic pathways and functions, based on gene ontology (GO) terms. The results showed an association of targets mostly with OMPs, such as OmpP2, OmpP2A2, MomP2, and OmpP2, among others (Figure 4B).

Combination of the results from TargetRNA2 and CopraRNA target predictors, indicated several cognate mRNA targets involved with various aspects of cell physiology and metabolism (Supplementary Table 2; Supplementary Figure 4). Among the predicted targets, mRNAs encoding the protease Lon (*lon* mRNA), and the ABC transporter complex MalEFGK (through identification of *malK* and *malE* mRNAs) were identified. We also identified genes encoding the proteins AccC, involved in fatty acid biosynthesis, AroQ, involved in chorismite metabolism, Asd, involved in aspartate metabolism, ZnuA, involved in zinc uptake, Ata, involved in cell adhesion, MetQ, involved in amino acid transport, and BioB, involved in biotin biosynthesis.

The genes encoding the SufE protein, associated with oxidative stress, and TolR protein, a component of Tol-Pal system, were also identified as potential targets of Rna01. Target prediction showed a common region of interaction of the targets in the Rna01 sequence, which allowed us to identify two putative seed regions in the Rna01 structure (seed regions 1 and 2; Supplementary Figures 4A, 5). STRING analysis of the software-predicted targets showed few protein associations and no functional enrichment (Supplementary Figure 4B). Rna01 may interact with different regions of the different targets, mostly with the 5′UTR, but also 3′UTR, or inside the coding sequences (Supplementary Figure 6).

### The expression of Rna01’s putative mRNA targets are affected by its absence

**Figure 5:**
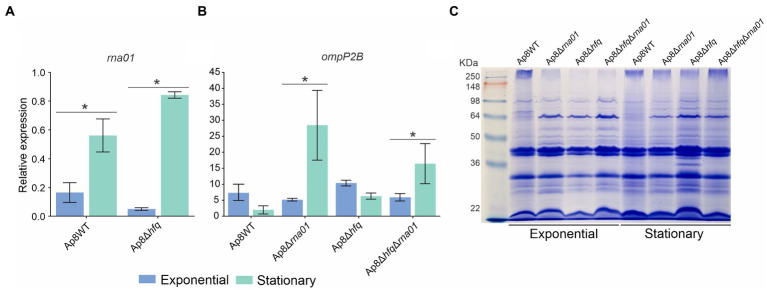
*rna01* and *ompP2B* expression and their effect in the outer membrane protein (OMP) contents. qPCR of *rna01*. **(A)** *ompP2B* **(B)** during exponential and stationary phases in the WT and mutant strains. **(C)** OMPs profile of the strains in exponential and stationary phases. *Significative difference by the *t*-test (*p* < 0.1). The experiment was performed in triplicate.

Analysis of the quantitative expression of *rna01* and predicted selected targets was performed by qPCR. Quantitative PCR of *rna01* showed that this sRNA was up-regulated during the stationary phase in Ap8WT and Ap8∆*hfq* strains (Figure 5A). However, *rna01* was down-regulated in the Ap8∆*hfq* strain during exponential growth (Figure 5A), suggesting that the stability of Rna01 is Hfq-dependent under this growth condition. Since seed region 2 was not conserved among homologs, and because of its distal location, it is less likely to be involved in target recognition, we focused on the targets that are more likely to bind the conserved seed region 1 for qPCR analysis. The putative target gene *ompP2B* had higher expression in the stationary phase in Rna01-deleted strains (*p* < 0.10; Figure 5B). The *ata_2* gene was down-regulated in mutant strains during the exponential phase (Supplementary Figure 7A), which correlates with reduced bacterial adhesion of the mutants in the stationary phase. The gene *tolR* (Tol-Pal system) was up-regulated in the double mutant strain during exponential growth, and in the mutant strains during the stationary phase (Supplementary Figure 7B). In contrast with *ompP2B*, the *ata_2* and *tolR* targets showed similar profiles of expression in the *rna01* and *hfq* mutants.

Outer membrane protein extraction revealed clear differences in the intensity of bands next to ~64 kDa between the WT and Ap8∆*hfq*, Ap8∆*rna01*, and Ap8∆*hfq*∆*rna01* strains, when adjudged by the presence and absence of protein bands. Minor differences were observed between exponential and stationary phases for the Ap8∆*hfq* strain (Figure 5C).

### Rna01 impacts several *Actinobacillus pleuropneumoniae* phenotypes

To evaluate the role of Rna01, mutant strains of Rna01 and/or Hfq were constructed. Gene replacements were confirmed by selecting the strains on trimethoprim (whose resistance is conferred by the newly added gene *dfrA14*), and by PCR for the presence of the sequence between *dfrA14* and *eriC* genes in the resulting ∆*rna*01 and Ap8∆*hfq*∆*rna*01 strains (Supplementary Figures 1A–D). Our strategy to replace the *rna01* gene by *dfrA14* did not affect the expression of the flanking genes *sbcB* and *eriC*, as detected by RT-PCR (Supplementary Figure 1E).

#### Rna01 impacts bacterial growth

**Figure 6:**
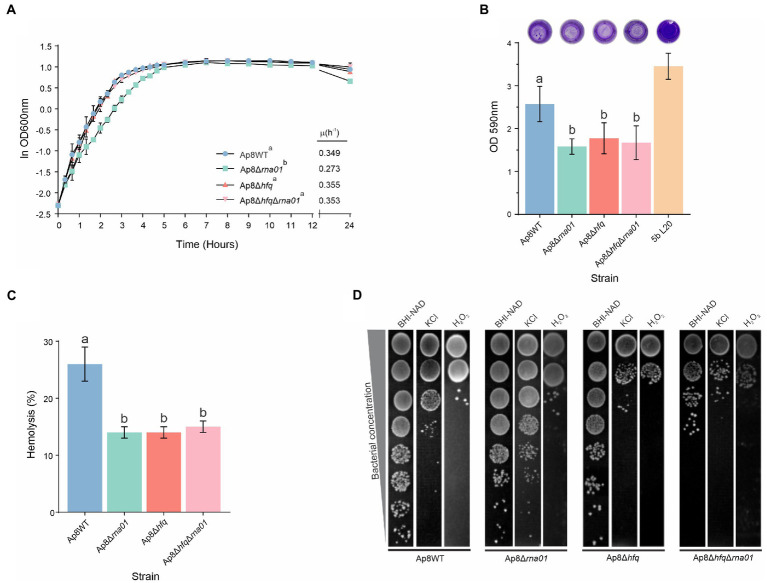
Phenotypic characterization of *Actinobacillus pleuropneumoniae rna01* mutant strains. **(A)** Bacterial growth in BHI-NAD at 37°C. Maximum growth rates (μ) are shown in the bottom-right corner. **(B)** Biofilm formation in polystyrene microtiter plates. The reference strain 5b L20 was used as positive control for biofilm formation. **(C)** Hemolytic activity on sheep blood agar. **(D)** Stress tolerance in KCl (0.1 M) and H_2_O_2_ (0.2 mM). Means with different letters are significantly different by the Tuckey’s test. The experiment was performed in triplicate.

Growth curves for the WT and mutant strains are presented in Figure 6A. No statistically significant difference (*p* > 0.05) in maximum growth rate (μ_max_) of the Ap8WT, Ap8∆*hfq* and Ap8∆*hfq*∆*rna*01 strains was found, although the μ_max_ for the ∆*rna*01 was lower than the others (*p* < 0.05; Figure 6A).

#### Rna01 impacts biofilm formation and hemolysis

All mutant strains, Ap8*∆rna01*, Ap8∆*hfq*∆*rna*01, and Ap8∆*hfq* had reduced biofilm-forming ability when compared to the Ap8WT and positive control (*p* < 0.05; Figure 6B). Unlike its derivatives, the Ap8WT strain also formed depositions of bacterial aggregates, while all mutant strains displayed uniform adherence in polystyrene wells (Figure 6B). All mutants displayed significantly impaired hemolytic activity (*p* < 0.05; Figure 6C). No additive effects on hemolysis were observed for the double mutant, Ap8∆*hfq*∆*rna*01.

#### Rna01 impacts susceptibility to stress

Upon osmotic stress with 0.1 M KCl, the strains Ap8∆*hfq* and Ap8∆*hfq*∆*rna*01 were more susceptible than the Ap8WT, while the strain Ap8*∆rna01* was less susceptible. Oxidative stress (H_2_O_2_) affected only *hfq*-deleted mutants, causing a growth reduction to these strains in comparison to the Ap8WT strain (Figure 6D). No strains were able to grow at 42°C, and no difference was observed in their susceptibility to ampicillin and tylosin (the MIC remained 4 μg/ml for all).

#### Rna01 and Hfq impact *Actinobacillus pleuropneumoniae* virulence against *Galleria mellonella*

**Figure 7:**
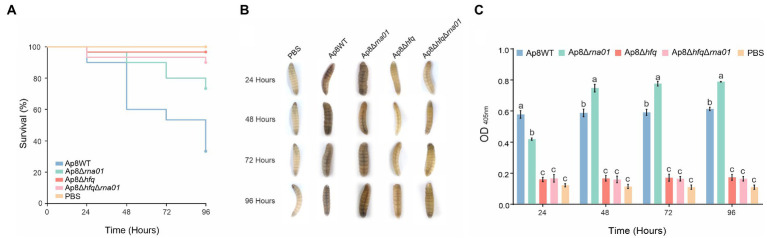
Effects of the deletion of the *rna01* gene on *Actinobacillus pleuropneumoniae* virulence. **(A)** Killing assay of *Galleria mellonella*; **(B)** Visual observation of larval melanization through the course of the experiment (only living larvae are shown, since dead larvae were black and dehydrated). **(C)** Optical density of larval hemolymph post-infection. Means with different letters are significantly different by the Tuckey’s test. The experiment was performed in triplicate.

To evaluate the virulence of strains lacking the *rna01* and/or *hfq* genes, we used *G. mellonella* as an infection model for virulence assays. Only ~34% of the *G. mellonella* larvae infected with the Ap8WT strain survived within 96 h post-infection (Figure 7A). Survival rates increased significantly, all above 70%, for the Ap8∆*hfq*, Ap8∆*rna*01, and Ap8∆*hfq*∆*rna*01 strains, as 72, 89, and 95% of the larvae infected, respectively, survived during the 96 h experiment (*p* < 0.05). The results obtained for the single mutants were statistically equivalent to those obtained for the double mutant strains (*p* < 0.05; Supplementary Table 3).

Visually, larvae infected with the negative control (PBS), the Ap8∆*hfq* and Ap8∆*hfq*∆*rna*01 strains showed no clear points of melanization (Figure 7B). However, quantitatively, the Ap8∆*hfq* and Ap8∆*hfq*∆*rna*01 strains induced a minor production of melanin, as their hemolymph was slightly more turbid than the negative controls (Figure 7C). The Ap8WT and Ap8∆*rna*01 strains, on the other hand, induced more melanization than the other strains (*p* < 0.05). The results in Figure 7C show that the Ap8∆*rna*01 strain, despite being attenuated in virulence, is capable of inducing an immune response in *G. mellonella* based on the melanization results. Dead larvae were completely melanized and dehydrated, thus no hemolymph was available for analysis.

### Rna01 impacts extracellular vesicle amounts, sizes, and toxicity

**Figure 8:**
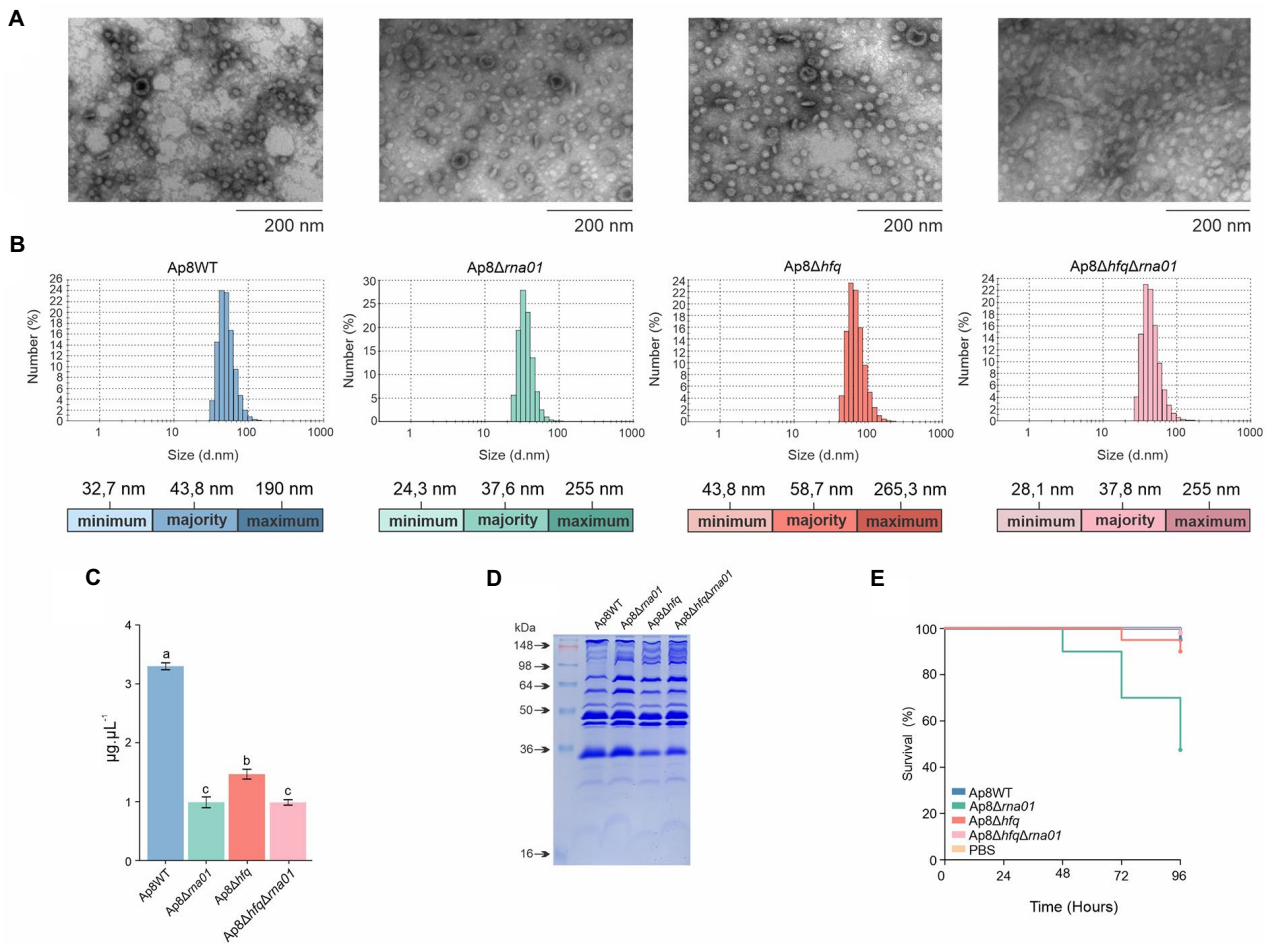
Rna01 and extracellular vesicle (EV) production by *Actinobacillus pleuropneumoniae*. **(A)** Transmission electron microscopy of EVs produced by *A. pleuropneumoniae* strains. **(B)** Size measurement by dynamic scattering light (DLS) of the EVs produced by the strains. Minimum, majority and maximum size of the EVs are shown by colored bars below each graph. **(C)** Relative abundance of EV production among strains. **(D)** OMP profile of EVs produced by the strains. **(E)** Killing assay of *G. mellonella* after administration of EVs. Means with different letters are significantly different by the Tuckey’s test. The experiment was performed in triplicate.

Transmission electron microscopy showing integrity of EVs (Figure 8A). EVs produced by the Ap8∆*hfq* strain were the largest, and those from *rna01*-mutant strains were the smallest, in comparison to WT (Figure 8B). All mutants produced less EVs than the WT (Figure 8C). The OMP profile revealed some changes in the intensity of bands next to ~64 kDa in the mutant strains compared to the Ap8WT strain (Figure 8D). However, EVs produced by the Ap8∆*rna01* strain were more toxic to *G. mellonella* than the others (*p* < 0.05; Figure 8E; Supplementary Table 4), although, no significant differences were observed in the melanization of the larvae (*p* > 0.05; Supplementary Figures 8A,B).

### Rna01 is a novel stress regulator

**Figure 9:**
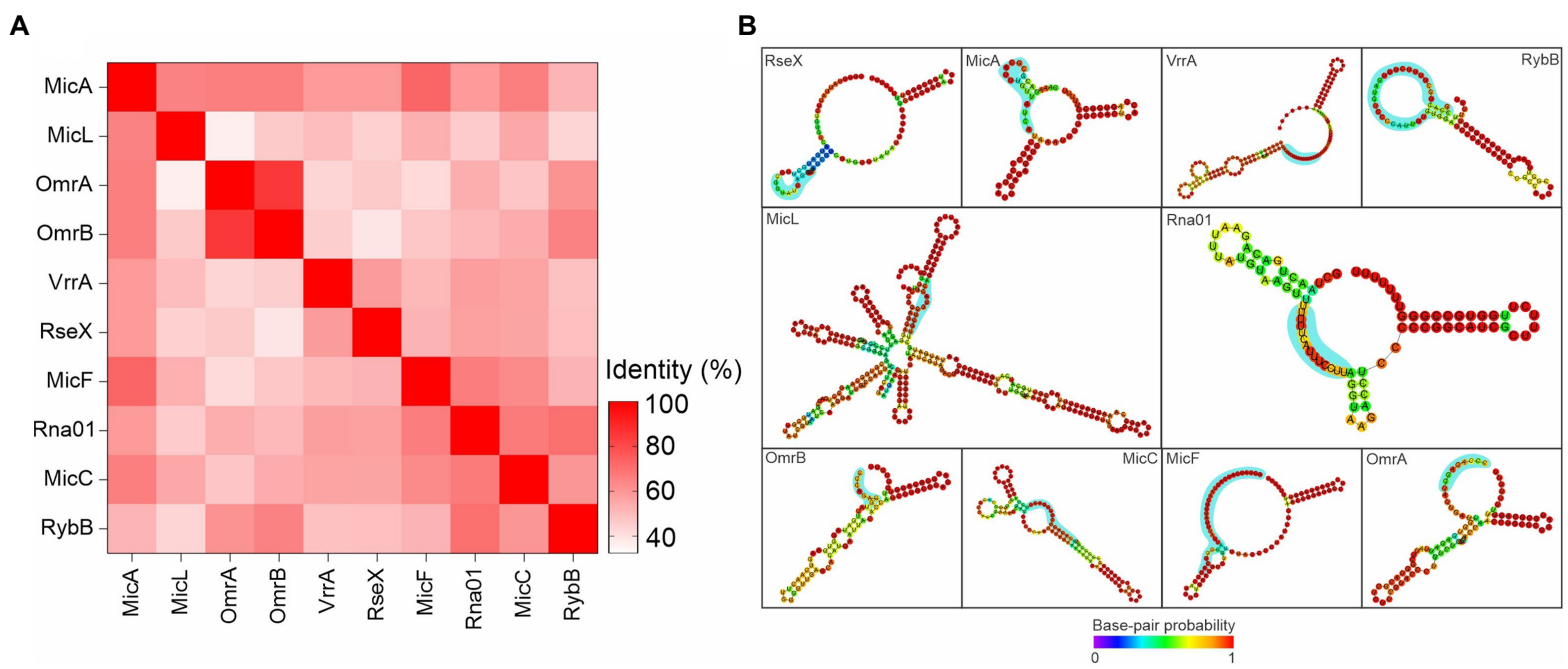
Comparative analysis among Rna01 and other extracytoplasmic stress associated sRNAs. **(A)** Heatmap based on matrix identity of the sRNAs. **(B)** Secondary structure of the sRNAs. The blue shades represent the seed region of interaction with OMP mRNA targets.

We investigated Rna01 as a possible novel sRNA associated with extracytoplasmic stress responses through comparisons with other sRNAs associated with the stress responses described in the literature. Results showed that Rna01 is not closely related to any other known sRNAs associated with bacterial extracytoplasmic stress (Figure 9A). However, Rna01 has a secondary structure similar to MicA (Figure 9B). Apart from Rna01, all stress response-associated sRNAs analyzed were absent in the *A. pleuropneumoniae* MIDG2331 genome.

## Discussion

In this work we have described, for the first time, the existence of sRNAs interacting with the RNA chaperone Hfq in *A. pleuropneumoniae*, the causative agent of porcine pleuropneumonia. Although this is the first study, as we are aware, to make a functional description of a sRNA in *A. pleuropneumoniae*, previous studies have shown the existence of other sRNAs in this bacterium. Our group, for example, described some molecules that were predicted by computational methods and confirmed experimentally (Rossi et al., 2016). Likewise, a study by Su et al. (2016) performed a deep RNA-seq to describe the transcriptome of the serovar 3 strain JL03 of this bacterium, leading to the discovery of novel sRNAs. Of the 14 sRNAs candidates identified here by co-IP/RNA-seq, eight have never been described before, according to our knowledge. Three were among the 17 of 23 computationally predicted sRNAs that were confirmed experimentally by our group before (Rossi et al., 2016). This work then expands the set of sRNAs identified in *A. pleuropneumoniae*. Although only approximately 50% of bacteria harbor the *hfq* gene (Orans et al., 2020), in those that do it can be an important RNA chaperone as demonstrated by *hfq* mutants of *A. pleuropneumoniae* being attenuated for virulence (Subashchandrabose et al., 2013; Pereira et al., 2015; Crispim et al., 2020). In some other bacteria, less widespread RNA chaperones, such as CsrA and ProQ, may play the same role of Hfq (Smirnov et al., 2016; Müller et al., 2019). These three chaperones are not mutually exclusive, for example, *Escherichia coli*, *Salmonella enterica, Bacillus subtilis,* and *Clostridium botulinum* have been described to encode combinations of two RNA chaperones (Quendera et al., 2020). Although the *proQ* and *csrA* genes are present in *A. pleuropneumoniae* genomes, there are no reports of the respective proteins acting as RNA chaperones for this species.

Additionally, not all sRNAs bind RNA chaperones to exert their activities, nor do all the Hfq-associated sRNAs depend on the chaperone to be protected from RNase degradation in the cell. In fact, growing evidence indicates that Hfq can not only bind sRNAs to (i) facilitate their interaction with mRNA cognates; (ii) protect them from; or (iii) lead them to degradation (Vogel and Luisi, 2011), but can also play additional regulatory roles. These include binding mRNAs alone to control their translation or modification, binding rRNAs and tRNAs to help with their maturation/processing, and affecting DNA conformation and compaction (Jiang et al., 2015; dos Santos et al., 2019). This likely explains why our co-IP was not only enriched with *trans-acting* RNAs, but also with two *cis*-acting RNAs, the FMN riboswitch and the S15 leader sequence.

The sRNA we chose to focus on, Rna01, is involved in stress response and virulence in *A. pleuropneumoniae*. Some of the targets predicted are related with the phenotypes observed for *A. pleuropneumoniae hfq* and *rna01* mutants. These include those obtained in this study, as well as previous data for *hfq* mutants showing reduced fitness and virulence in pigs and *G. mellonella*, and impaired capacity to respond to different types of stress (Subashchandrabose et al., 2013; Pereira et al., 2015; Crispim et al., 2020). Because the lack of Hfq can lead to different phenotypes in different serovars of *A. pleuropneumoniae* (Crispim et al., 2020), and targets are included in a complex and integrated network, it is complex to define which Hfq-sRNA-mRNA interactions will lead to a certain phenotype. Additionally, approximately 10% of the protein-coding genes from *A. pleuropneumoniae* serovar 8 strains still have unknown functions (Prado et al., 2020).

Although *A. pleuropneumoniae* virulence is highly attributed to the serovar-associated pattern of production of RTX toxins (Frey, 2011), different strains of the same serovar can exhibit varying degrees of virulence, since other features may contribute to the outcome of infection (Pereira et al., 2015, 2018). Thus, the activities conferred by Hfq, Hfq-dependent and -independent sRNAs, add extra layers of complexity to this bacterium’s virulence.

*In silico* analysis of Rna01 also showed putative σ^E^ and σ^70^ promoters and typical Hfq binding sites, with minor differences among homologs (Figure 3; Supplementary Figure 2). The search for homologs for this sRNA showed a wide distribution among members of the *Pasteurellaceae* family, being mostly present in related veterinary pathogens, but also the human pathogen *H. ducreyi*, which may reflect the importance of this sRNA among *Pasteurellaceae* members in different environments (Supplementary Figure 3).

Although it was not possible to identify the abundance of Rna01 by Northern blotting analysis in the *hfq* mutant strain (Figure 2A), Rna01 was upregulated in stress (stationary phase) but downregulated in non-stressful (exponential phase) conditions as observed in the qPCR analysis (Figure 5A). This suggests that Rna01 is dependent on Hfq in some, but not all, conditions for action and stability.

Some of the phenotypes presented by the *rna01* mutant matched those of the *hfq* mutant, and were not additive in the double mutant strain. This infers that, in the absence of Hfq, Rna01 may also be absent, possibly due to RNase degradation. Biofilm formation contributes to pathogenicity (Hathroubi et al., 2018), and the hemolysis observed is related to the expression of the RTX proteins. As the mutant strains showed reduced biofilm formation and hemolytic activity (Figure 6), we evaluated the virulence of these strains in *G. mellonella*, an excellent surrogate infection model predictive of virulence found in the natural host—the pig. We found significant differences between the Ap8∆*hfq* and ∆*rna*01 strains observed here related to the stimulation of *G. mellonella*’s immune response (Figure 7). Melanization is an essential and readily observed characteristic of the larva’s humoral response against microorganisms, resulting in synthesis and deposition of melanin around the invading pathogen (Pereira et al., 2020). Here we observed that, even though all mutations reduced larval killing, melanization was still observed for the ∆*rna*01 mutant strain, but not the *hfq* or double mutant (Figure 7). Interestingly, for osmotic stress, Ap8∆*rna*01 was less sensitive than the WT strain, while the opposite was observed for the Ap8∆*hfq* and double mutant. This indicates that some phenotypes of *A. pleuropneumoniae* are independent of Rna01 (as seen for osmotic stress) but influenced by the global regulator Hfq.

Although the interactions of Rna01 with its predicted targets remain to be confirmed experimentally, some of the targets predicted may be responsible for the phenotypes observed in this work and are corroborated by previous studies. A previous study (Xie et al., 2016), for example, showed that a *lonA* mutant (annotated as *lon*) in MIDG2331, displayed reduced biofilm formation, stress tolerance and pathogenicity. However, the Ap8∆*rna01* strain showed higher tolerance to osmotic stress in relation to the other strains, although the same was not observed in the other phenotypic analyses. Another predicted Rna01 target, *sufE*, showed reduced tolerance to oxidative and acid stress in *E. coli* (Lee et al., 2010). Likewise, Rna01’s predicted targets *malK* and *malE* are known to regulate *malT* in *E. coli* (Böhm et al., 2002). A study with *A. pleuropneumoniae* showed that knocking out the *malT* gene led to reduction in growth rate (Lone et al., 2009). These results suggest that the interaction of Rna01 with these predicted targets are worthy of investigation, with a potential to affect the fitness of *A. pleuropneumoniae*.

Studies with an *aroQ* mutant showed an attenuated phenotype for *A. pleuropneumoniae* (Ingham et al., 2002). Analysis of a *znuA* mutant showed attenuation in experiments with animals for *Brucella abortus* and the members of the *Pasteurellaceae* family *Haemophilus ducreyi*, *Pasteurella multocida* and *A. pleuropneumoniae* (Yang et al., 2006; Yuan et al., 2014).

Despite the absence of studies involving *tolR* and *metQ* in *A. pleuropneumoniae,* deletion of homologues of these predicted Rna01 targets in *Edwardsiella ictaluri* (Abdelhamed et al., 2016) and *Streptococcus pneumoniae* (Basavanna et al., 2013), resulted in attenuation. Studies with *Acinetobacter baumanii* reported that the absence of the adhesin Ata strongly affects the virulence and adhesion of this species (Weidensdorfer et al., 2019). Also, studies with *Salmonella enterica* serovar Typhimurium showed attenuation phenotypes in mutants for the *bioB* and *asd* genes (Piao et al., 2010; Denkel et al., 2013).

Investigation of conserved regions of the Rna01 sequence revealed a fragment in a single-strand region of the secondary structure of Rna01 predicted to interact with outer membrane associated genes (Supplementary Figure 3). Previous studies reported that seed regions need to be unstructured and base pairing regions are commonly single strands, as previously reviewed (Updegrove et al., 2015).

The stress sigma factor σ^E^ promoter and the targets predicted indicate that Rna01 might be associated with post-transcriptional regulation of diverse genes in a stress response condition, mostly extracytoplasmic stress. These findings are consistent with some previous reports, e.g., *S.* Typhimurium, in which σ^E^ sRNAs, such as MicA and RybB, respond to membrane stress (Papenfort et al., 2006). The well-studied MicA and RybB sRNAs are responsible for alleviating stress and mediating interconnection with the envelope stress network. These sRNAs target *omp* mRNAs predominantly, but also some non-*omp* targets (Klein and Raina, 2017).

Like other well studied sRNAs associated with stress responses, such as MicA, RybB, and VrrA, Rna01 may interact, by base-pairing next to the ribosomal binding site (RBS), with diverse genes that code for OMPs, which is commonly associated with down-regulation of these targets in extracytoplasmic stress conditions (Pfeiffer et al., 2009).

Analysis focused on investigating Rna01 as an extracytoplasmic stress associated sRNA showed strong evidence of this function, as seen in the qPCR analysis, as this sRNA is up-regulated during stationary growth (a stressful condition; Figure 5A). Also, the same experiment showed that Rna01 is expressed in the absence of Hfq during the stationary phase, but seems to be unstable in *hfq* mutants in a non-stressful condition (Figure 5A). By investigating expression of *ompP2B*, a putative Rna01 target, we found that, in the absence of Rna01 in the stationary phase, this target had increased expression (Figure 5B), which is consistent with the hypothesis that Rna01 acts by inhibiting the translation of OMPs by blocking RBS sequences. The effect of Rna01 on OMP expression was also observed by SDS-PAGE and Coomassie blue staining of OMP preparations (Figure 5C). Although we hypothesized that Rna01 directly blocks the RBS of the OMPs mRNAs, it is also possible that differences in the OMP profiles is affected by the periplasmic serine protease DegP, encoded by the *degP* gene, another predicted Rna01 target. Interestingly, the Ap8∆*hfq* and Ap8∆*hfq*∆*rna*01 strains also showed differences in the OMP profiles, compared to the Ap8WT strain, which may be explained by the pleiotropic effect of the Hfq chaperone (Vogel and Luisi, 2011). Curiously, for the *ata_2* and *tolR* targets, the strains lacking Rna01 or Hfq showed similar expression, which may result from Hfq-dependence (Supplementary Figure 7). This may be explained considering that the seed region of Rna01 that interacts with OMPs targets are in a single strand (considering the native secondary structure predicted here) and may not depend on Hfq for this interaction. However, the seed region of Rna01 that is predicted to interact with other targets is located in a double strand portion of Rna01, where the free energy is higher and may depend on Hfq, which structures the sRNA sequence to form the complex sRNA-Hfq-mRNA (Updegrove et al., 2016).

As Rna01 showed clear evidence of being associated with OMP regulation, we also investigated its effect on EVs (also called outer membrane vesicles—OMVs). There was less production of the EVs by mutants lacking Rna01, and these were smaller and of higher toxicity for *G. mellonella* (Figure 8). A similar study with *Vibrio cholerae* revealed that VrrA, a stress response associated sRNA, which also regulates OMPs expression, affects EV production (Song et al., 2008).

**Figure 10:**
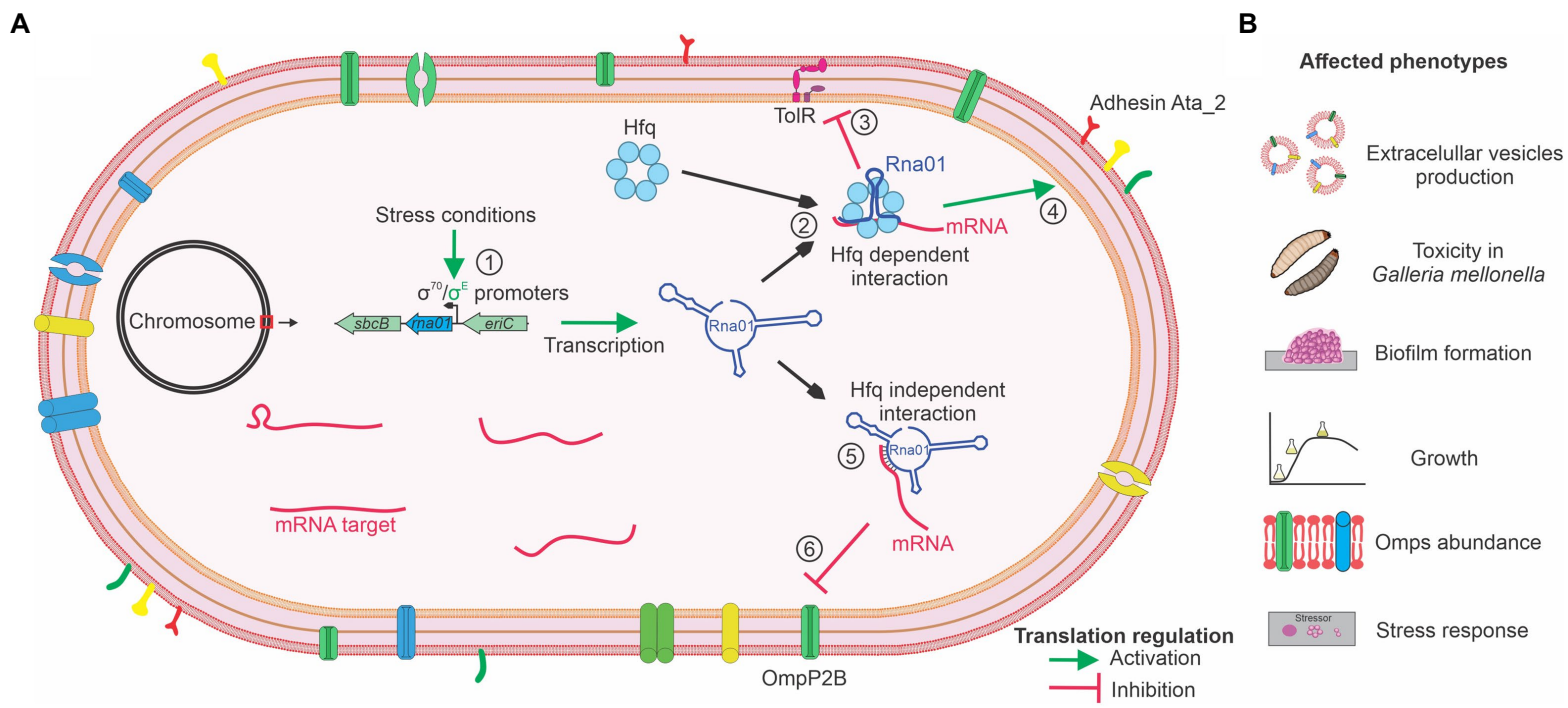
Mechanism of activity proposed for the novel sRNA Rna01. **(A)** The Figure summarizes the expression of *rna01* in normal and stress conditions (by σ^70^ and σ^E^, respectively) (1), followed by regulation of the targets in an Hfq dependent manner (2), inhibiting (3), or inducing (4) translation of the targets. Also, Rna01 regulates translation of the OMP targets in a Hfq-independent manner (5) by inhibiting translation (6). **(B)** The activity of Rna01, mediated or not by the chaperone Hfq, affects diverse phenotypes of *A. pleuropneumoniae*.

Based on the results presented in this study regarding Rna01 and predicted target(s) expression, the phenotypes affected and dependence of Hfq, we propose a mechanism of activity for this sRNA which is summarized in Figure 10. Considering that other sRNAs (MicA, MicF, MicC, MicL, OmrA, OmrB, VrrA, RseX, and RybB), associated with the stress response in other species, were not found in the *A. pleuropneumoniae* genome, and that comparative analysis with these sRNAs showed Rna01 as a different sRNA, altogether, these results suggest that Rna01 is an sRNA associated with the extracytoplasmic stress response of *A. pleuropneumoniae*.

In conclusion, this work is a step forward in the understanding of the influence of sRNAs in the physiology and virulence of *A. pleuropneumoniae*, which our studies indicate are multilayered and complex, and are greatly influenced by the molecular chaperone Hfq and associated RNAs. Also, this study reports, for the first time, an *A. pleuropneumoniae* sRNA (Rna01) that is associated with extracytoplasmic stress, virulence and EV production. Further analyses of sRNAs, such as those we have identified as being Hfq-dependent in our co-IP experiments, are worthy of study to further elucidate the molecular basis of gene regulation and virulence of *A. pleuropneumoniae*.

## Data availability statement

The datasets presented in this study can be found in online repositories. The names of the repository/repositories and accession number(s) can be found in the article/Supplementary material.

## Author contributions

All authors helped conceiving the study. GS, CR, JR, NS, JB, YL, AW, KG, PF, and DB produced the data. GS, CR, JR, JB, and DC analyzed the data. JB, AC, DB, and PL coordinated the study. GS, CR, JR, JB, DB, and PL wrote the paper. All authors contributed to the article and approved the submitted version.

## Funding

The authors thank CNPq (201840/2011-1, 407849/2012-2, 142495/2014-0, and 141328/2018), FAPEMIG (APQ-01586-18; APQ-00772-19; APQ-01433-22), CAPES/PROEX (23038.019105/2016-86 and 23038.002486/2018-26), FINEP (Núcleo de Microscopia e Microanálise–UFV), BBSRC (BB/K021109/1, BB/G019177/1, BB/M023052/1, BB/M020576/1, BB/S020543/1, BB/P001262/1, and BB/G018553), and CONFAP—the UK Academies (CBB-APQ-00689-16).

## Conflict of interest

The authors declare that the research was conducted in the absence of any commercial or financial relationships that could be construed as a potential conflict of interest.

## Publisher’s note

All claims expressed in this article are solely those of the authors and do not necessarily represent those of their affiliated organizations, or those of the publisher, the editors and the reviewers. Any product that may be evaluated in this article, or claim that may be made by its manufacturer, is not guaranteed or endorsed by the publisher.
