# Supplementary material for: Identification of small RNAs associated with RNA chaperone Hfq reveals a new stress response regulator in Actinobacillus pleuropneumoniae
Source: Front Microbiol. 2022 Oct 4;13:1017278. doi: 10.3389/fmicb.2022.1017278 (PMC9577009; doi:10.3389/fmicb.2022.1017278)
Supplement: Supplementary file 1 [file Data_Sheet_1.docx]

Supplementary Material

| 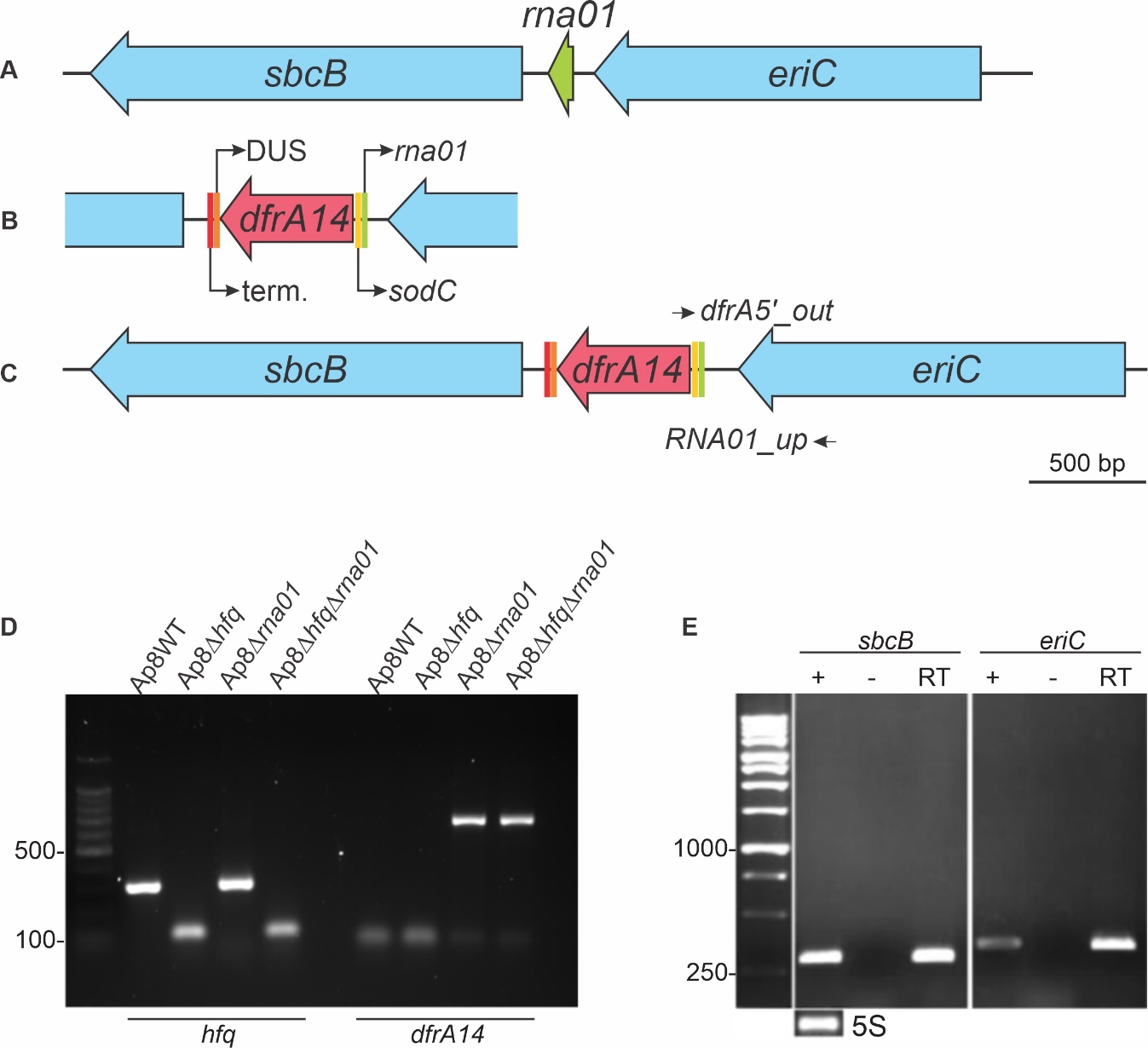 |
| --- |
| **Supplementary Figure 1.** Construction and confirmation of *A. pleuropneumoniae* Rna01 mutant strains. The original sequences of the *rna01* gene in MIDG2331 (WT) and Ap8∆*hfq* strains **(A)** were replaced by a cassette containing the trimethoprim resistance gene *dfrA14*, controlled by the *sodC* promoter sequence, followed by a DNA uptake sequence (DUS) and a terminator (term.) **(B),** flanked by 500 bp of the genes *scbB* and *eriC* **(C)*.*** The Rna01 and *hfq* single and double mutants were confirmed by PCR to detect the absence of *hfq* and presence of *dfrA14*, in the corresponding strains **(D)**. The maintenance of expression of *rna01*’s flanking genes *sbcB* and *eriC* in the ∆*rna01* strains (RT) was verified by RT-PCR **(E)** Total DNA free-RNA isolated from the WT strain subjected to cDNA synthesis of untreated were used as positive (+) and negative (-) controls. |


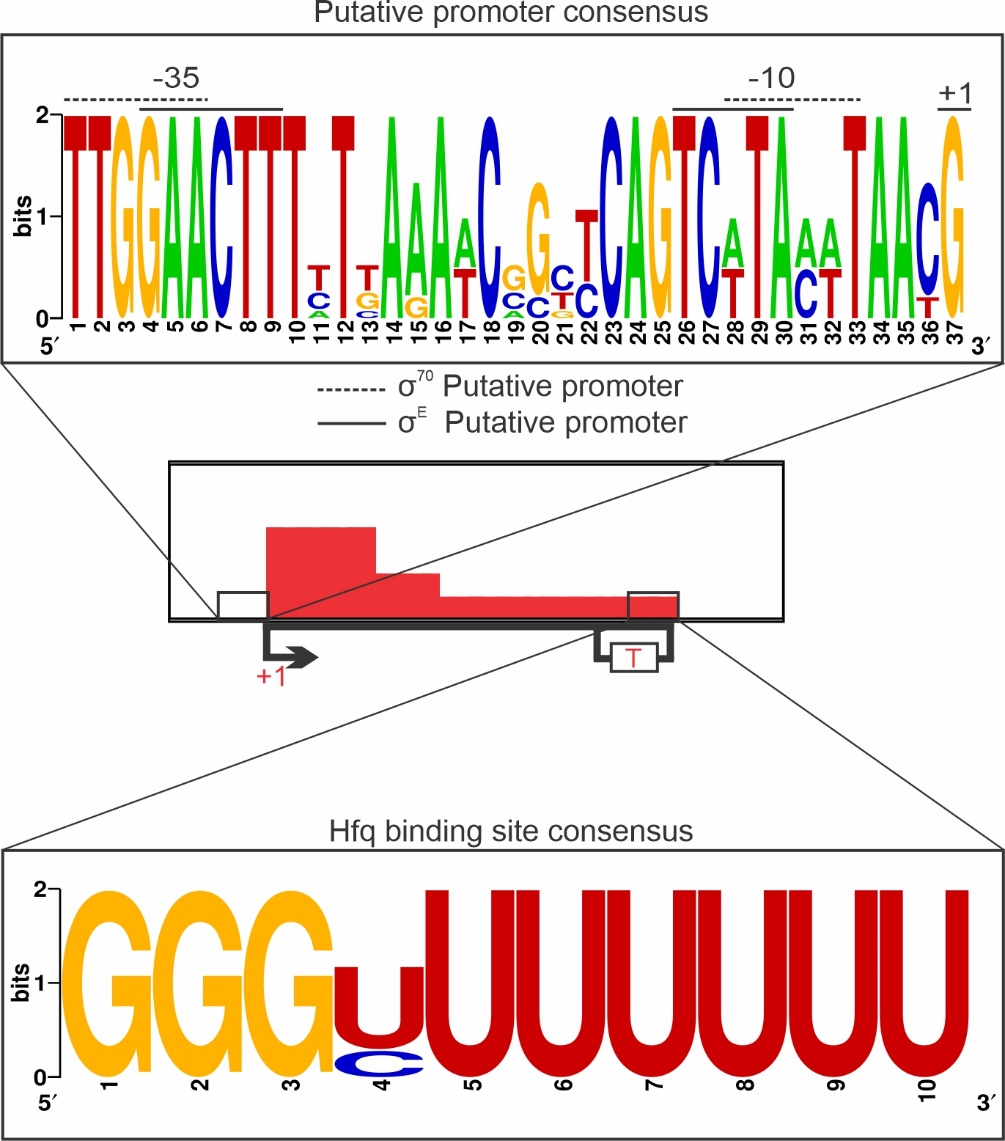


**Supplementary Figure 2.** Analysis of Rna01 homologues. (B) Conservation of Rna01’s putative Hfq-binding site and promoters regions.


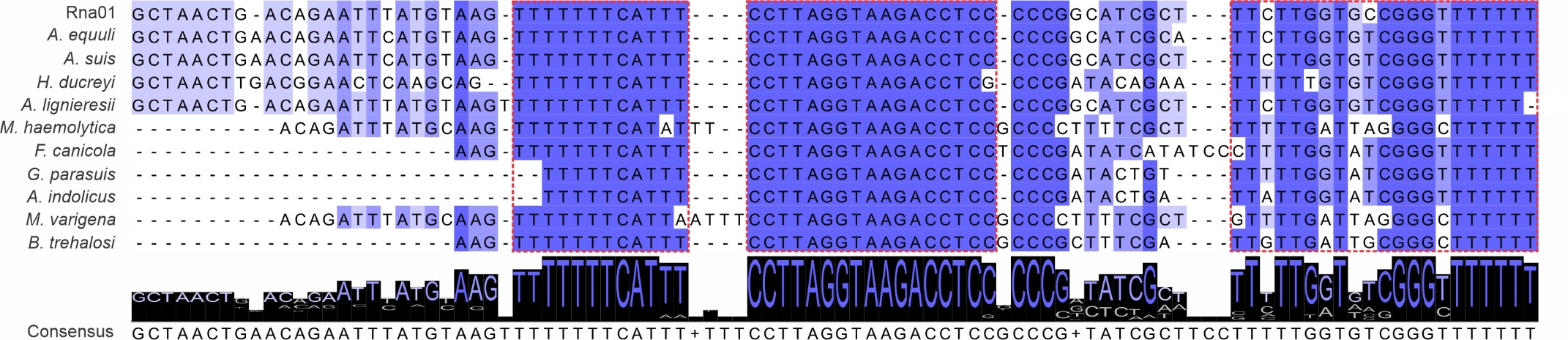


**Supplementary Figure 3.** Alignment of Rna01 homologues sequences. Alignment of homologues was developed using Jalview. Conserved regions are highlighted with red dotted lines and the consensus sequence is showed at bottom.

Supplementary Table 1. Potential mRNA targets of the Rna01 manually predicted in *Actinobacillus pleuropneumoniae*.

| **Target** | **Locus on MIDG2331** | **Energy (kcal/mol)** | **Description** |
| --- | --- | --- | --- |
| MIDG2331_00243 | MIDG2331_00243 | -10.78 | putative lipoprotein |
| *nrfB* | MIDG2331_00104 | -5.14 | nitrate reductase, cytochrome-C type protein |
| *omlA* | MIDG2331_01204 | -4.92 | outer membrane lipoprotein A precursor |
| *comEA* | MIDG2331_01571 | -6.16 | DNA uptake protein-binding protein |
| *phoR_2* | MIDG2331_01297 | -6.24 | phosphate regulon sensor protein |
| *phoB* | MIDG2331_01298 | -7.63 | phosphate regulon transcriptional regulatory protein |
| *rpoH* | MIDG2331_02050 | -5.92 | RNA polymerase sigma-32 factor |
| MIDG2331_00952 | MIDG2331_00952 | -9.15 | TPR repeat-containing protein |
| *exbD2* | MIDG2331_00080 | -5.99 | biopolymer transport protein |
| *potD2* | MIDG2331_00385 | -7.97 | spermidine/putrescine-binding periplasmic protein |
| *lsgD* | MIDG2331_01068 | -7.79 | glycosyltransferase involved in LPS biosynthesis |
| *lolB* | MIDG2331_00799 | -5.47 | outer membrane lipoprotein LolB |
| *acrA2* | MIDG2331_00863 | -6.2 | membrane-fusion protein |
| *lpp* | MIDG2331_00037 | -6.56 | outer membrane lipoprotein |
| MIDG2331_01099 | MIDG2331_01099 | -6.3 | NirD/YgiW/YdeI family stress tolerance protein |
| *ompD* | MIDG2331_00430 | -6.99 | outer membrane protein D-15 |
| *degP* | MIDG2331_01359 | -6.91 | periplasmic serine protease |
| *tadE* | MIDG2331_00535 | -10.46 | tight adherence protein E |
| *ompP2B* | MIDG2331_00658 | -9,09 | outer membrane protein P2-like protein |
| MIDG2331_00918 | MIDG2331_00918 | -6.92 | YdgA family protein |
| *lamB* | MIDG2331_01276 | -8,96 | maltoporin |
| MIDG2331_01616 | MIDG2331_01616 | -9.41 | thioredoxin-like protein |
| *fhuA* | MIDG2331_02245 | -9,66 | outer membrane ferric hydroxamate receptor |
| *ompW* | MIDG2331_01124 | -12.55 | outer membrane protein ompW precursor |
| *momP2* | MIDG2331_02079 | -6.98 | major outer membrane protein |
| *rbsB1* | MIDG2331_01591 | -10.54 | periplasmic sugar-binding protein |
| *dsbE2* | MIDG2331_01091 | -10.06 | putative thiol:disulfide interchange protein |
| *cas1f* | MIDG2331_00220 | -9.27 | CRISPR-associated protein |
| *rbsD* | MIDG2331_01859 | -10.57 | high affinity ribose transport protein |
| *ompP2A2* | MIDG2331_00006 | -11.76 | outer membrane protein P2 |

Supplementary Table 2. Potential mRNA targets of the Rna01 predicted by TargetRNA2 and CopraRNA in *Actinobacillus pleuropneumoniae.*

| **TargetRNA2** | | | | |
| --- | --- | --- | --- | --- |
| **Target** | **Locus on MIDG2331** | **Energy** | **P-value** | **Description** |
| *lon* | MIDG2331_RS01940 | -19.62 | 0.000 | Lon protease |
| *rarD* | MIDG2331_RS05305 | -13.5 | 0.003 | Thiosulfate sulfurtransferase |
| *ppiB* | MIDG2331_00946 | -10,77 | 0,016 | Peptidyl-prolyl cis-trans isomerase |
| *murQ* | MIDG2331_RS08355 | -10.41 | 0.019 | N-acetylmuramic acid 6-phosphate etherase |
| *dppA2* | MIDG2331_00067 | -10,15 | 0,022 | ABC transporter substrate-binding protein |
| *xseB* | MIDG2331_RS04060 | -9.74 | 0.027 | Exodeoxyribonuclease 7 small subunit |
| *fhuA* | MIDG2331_02245 | -9,66 | 0,028 | TonB-dependent siderophore receptor |
| *malK* | MIDG2331_RS06255 | -9.58 | 0.029 | Maltose/maltodextrin import ATP-binding protein MalK |
| *cysQ* | MIDG2331_RS06740 | -9.57 | 0.029 | 3'(2'),5'-bisphosphate nucleotidase CysQ |
| *cas1f* | MIDG2331_RS01085 | -9.27 | 0.033 | CRISPR-associated endonuclease Cas1 |
| *exbB1* | MIDG2331_01754 | -9,13 | 0,035 | MotA/TolQ/ExbB proton channel family protein |
| *ompP2B* | MIDG2331_00658 | -9,09 | 0,036 | porin |
| *tolR* | MIDG2331_RS01555 | -9.05 | 0.036 | Tol-Pal system protein TolR |
| *lamB* | MIDG2331_01276 | -8,96 | 0,038 | maltoporin |
| *rplR* | MIDG2331_RS09745 | -8.89 | 0.039 | 50S ribosomal protein L18 |
| *ispC* | MIDG2331_RS02090 | -8.79 | 0.041 | 1-deoxy-D-xylulose 5-phosphate reductoisomerase |
| *purE* | MIDG2331_RS03280 | -8.77 | 0.041 | N5-carboxyaminoimidazole ribonucleotide mutase |
| *moeB* | MIDG2331_RS01610 | -8.69 | 0.042 | Molybdopterin-synthase adenylyltransferase |
| **CopraRNA** | | | | |
| **Target** | **Locus on MIDG2331** | **Fdr** | **P-value** | **Description** |
| *malE* | MIDG2331_RS06260 | 0,090615546 | 0,000183598 | maltose/maltodextrin ABC transporter substrate-binding protein MalE |
| *bioB* | MIDG2331_RS00605 | 0,090615546 | 0,000186356 | biotin synthase |
| *accC* | MIDG2331_RS10255 | 0,180388642 | 0,000463724 | acetyl-CoA carboxylase biotin carboxylase subunit |
| *aroQ* | MIDG2331_RS10240 | 0,186751572 | 0,000714664 | 3-dehydroquinate dehydratase |
| *pssA* | MIDG2331_RS00215 | 0,186751572 | 0,000779764 | phosphatidylserine synthase |
| *hemD* | MIDG2331_RS05130 | 0,186751572 | 0,000942524 | uroporphyrinogen-III synthase |
| *yedF* | MIDG2331_RS10805 | 0,186751572 | 0,001027049 | SirA-like protein |
| *ubiD* | MIDG2331_RS07875 | 0,186751572 | 0,001056179 | 4-hydroxy-3-polyprenylbenzoate decarboxylase |
| *mtgA* | MIDG2331_RS02450 | 0,273161438 | 0,001966201 | monofunctional biosynthetic peptidoglycan transglycosylase |
| *asd* | MIDG2331_RS00025 | 0,323193418 | 0,002658661 | aspartate-semialdehyde dehydrogenase |
| *lctP* | MIDG2331_RS02310 | 0,337316752 | 0,00295043 | L-lactate permease |
| *plpB* | MIDG2331_RS04595 | 0,337316752 | 0,003282774 | methionine ABC transporter substrate-binding protein MetQ |
| *znuA* | MIDG2331_RS07880 | 0,337316752 | 0,003295125 | zinc ABC transporter substrate-binding protein |
| *sufE* | MIDG2331_RS06570 | 0,368974332 | 0,004190709 | Derived by automated computational analysis using gene prediction method: Protein Homology. |
| *argB* | MIDG2331_RS01235 | 0,368974332 | 0,004363193 | acetylglutamate kinase |
| *fdnI* | MIDG2331_RS04515 | 0,409768106 | 0,005056264 | formate dehydrogenase subunit gamma |
| *murF* | MIDG2331_RS00070 | 0,455255302 | 0,006085674 | UDP-N-acetylmuramoyl-tripeptide--D-alanyl-D- alanine ligase |
| *mltA* | MIDG2331_RS04100 | 0,457230577 | 0,006379401 | murein transglycosylase A |
| *hybE* | MIDG2331_RS06855 | 0,457230577 | 0,006731636 | hydrogenase |
| *sucD* | MIDG2331_RS02330 | 0,457230577 | 0,006817319 | succinate--CoA ligase subunit alpha |
| *pheT* | MIDG2331_RS02930 | 0,492356498 | 0,008100466 | phenylalanine--tRNA ligase subunit beta |


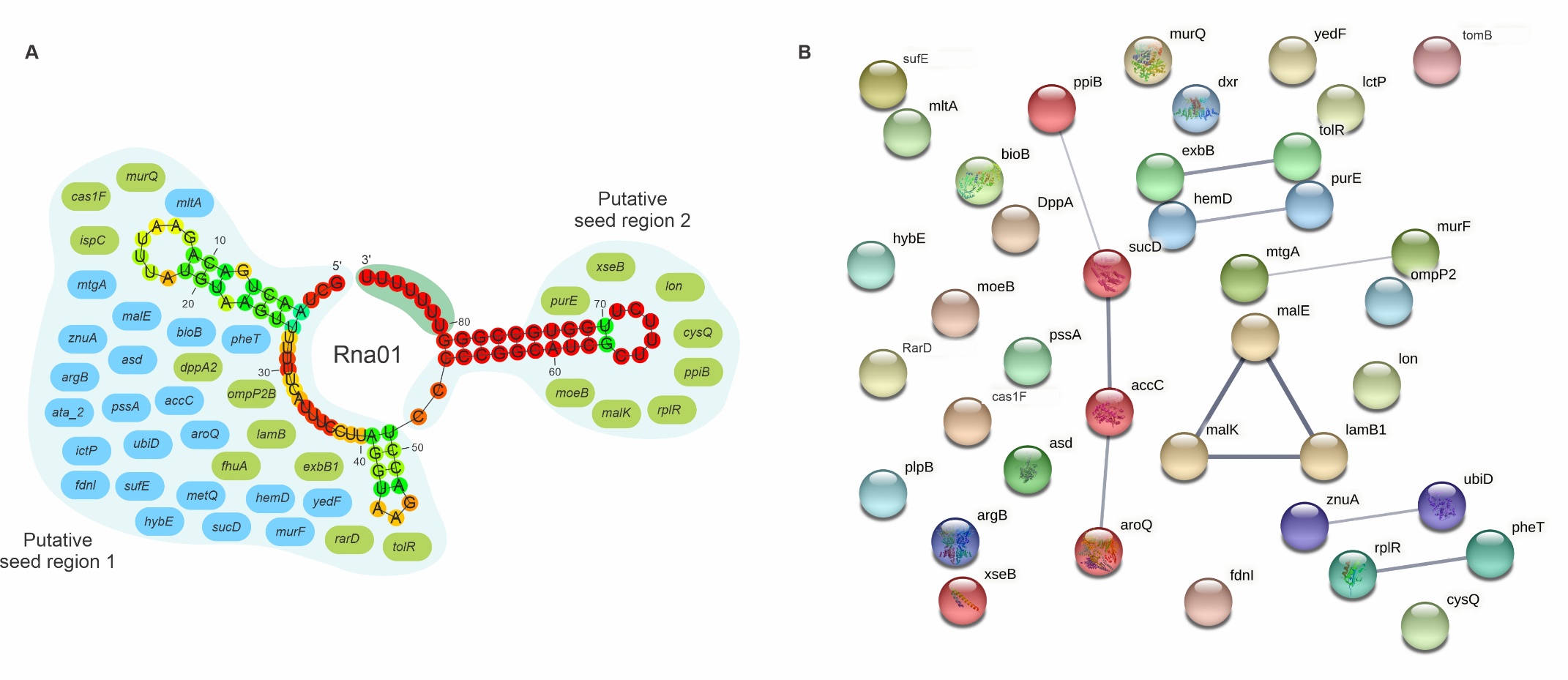


**Supplementary Figure 4.** Investigation of targets prediction for Rna01. **(A)** targets predicted for Rna01 using CopraRNA and TargetRNA2. Putative regions of interaction (seed regions 1 and 2) are showed in the figure. Targets predicted by TargetRNA2 are shown in green and targets predicted by CopraRNA, in blue. Nucleotides circled in dark green represent the Hfq binding site **(B)** protein-protein association of targets predicted for Rna01. Grey lines represent protein-protein associations, and their thickness indicates the strength of data support. Associations are meant to be specific and meaningful, for example, proteins that jointly contribute to a shared function. Genes were analyzed with a moderate confidence 0.400 and Markov clustering method (MCL) with inflation parameter 1.1. Associated proteins share color of the network.


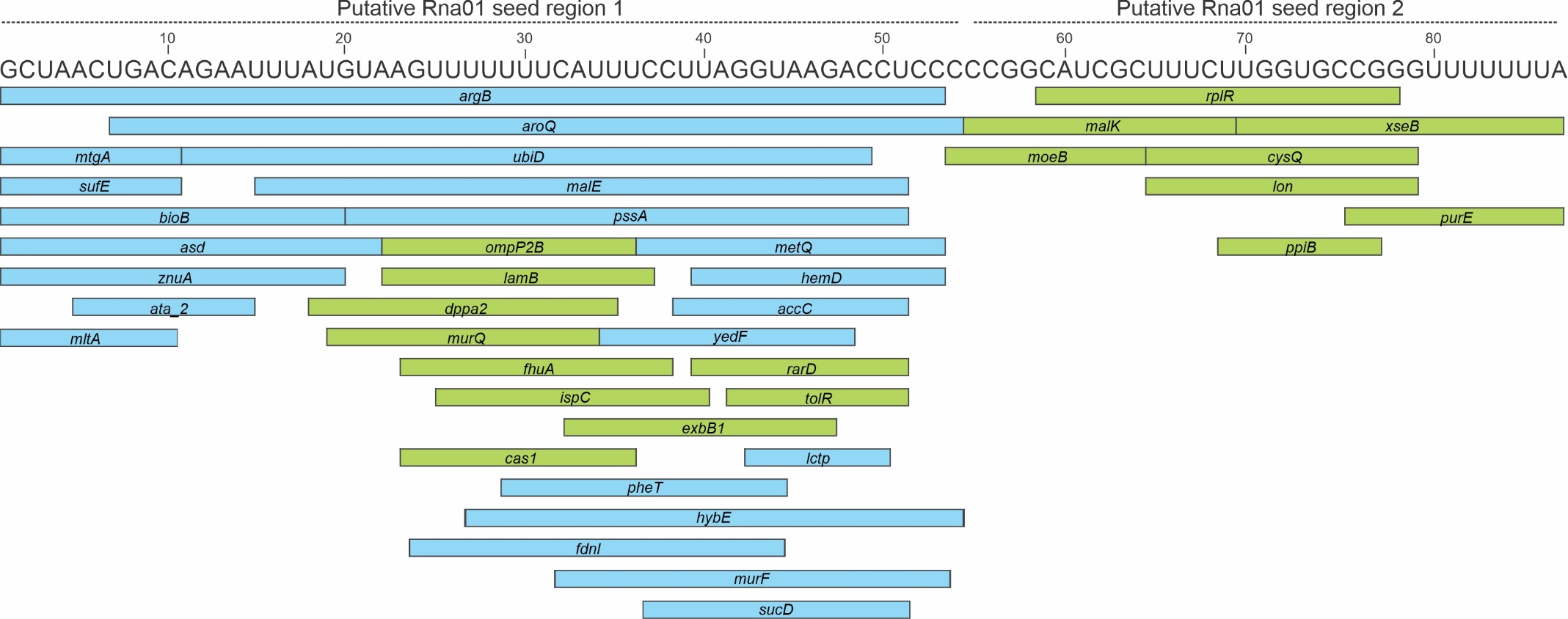


**Supplementary Figure 5.** Putative “seed” region of Rna01’ targets. Target prediction by TargetRNA2 and CopraRNA shows the putative region of interaction between Rna01 and its targets. Targets predicted by TargetRNA2 are shown in green and targets predicted by CopraRNA, in blue.


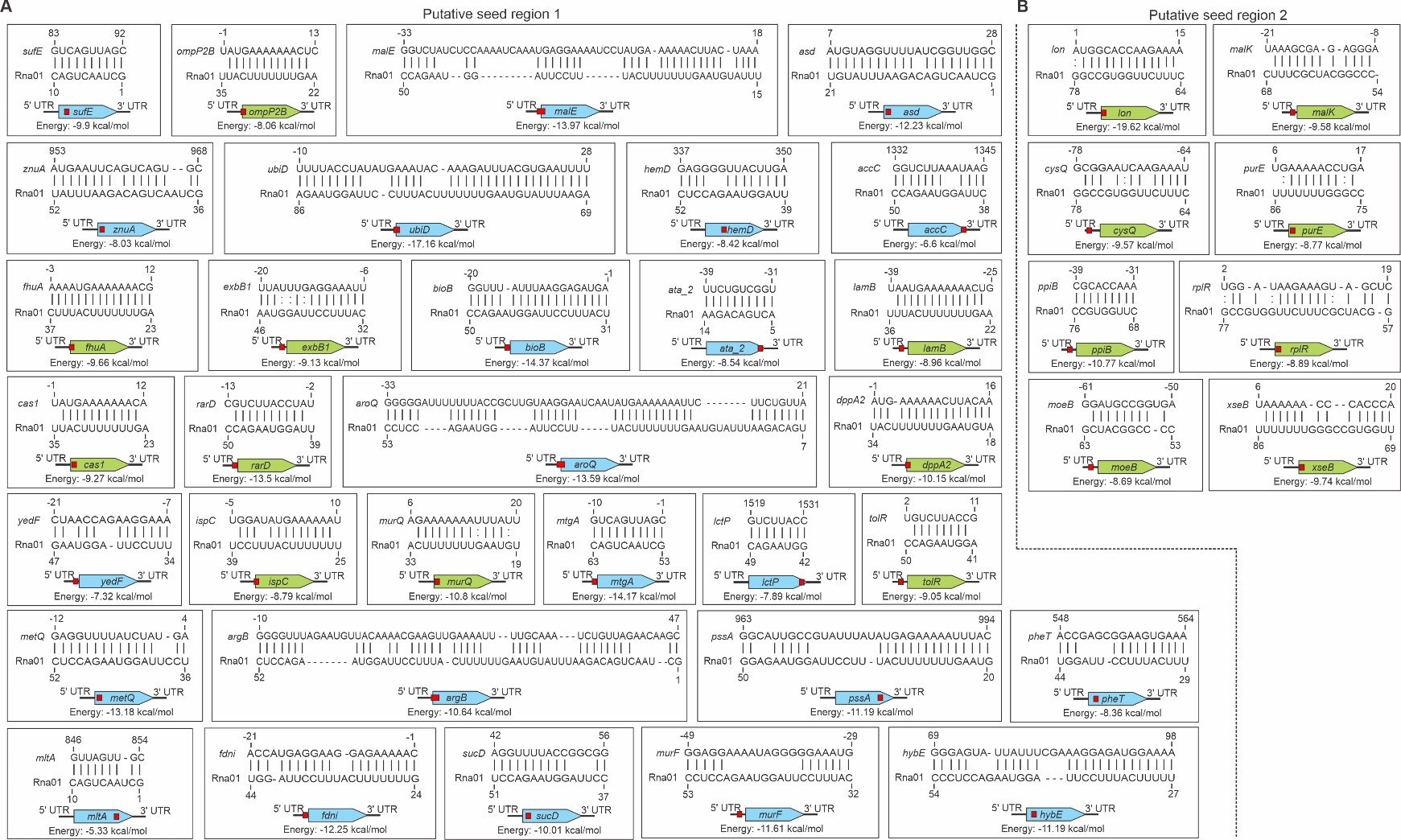


Supplementary Figure 6 | RNA-RNA interaction between Rna01 and respective targets. Putative region of interaction of the seed region 1 (A) and seed region 2 (B) of the Rna01 and the mRNAs targets. Targets predicted by TargetRNA2 are shown in green and targets predicted by CopraRNA, in blue.


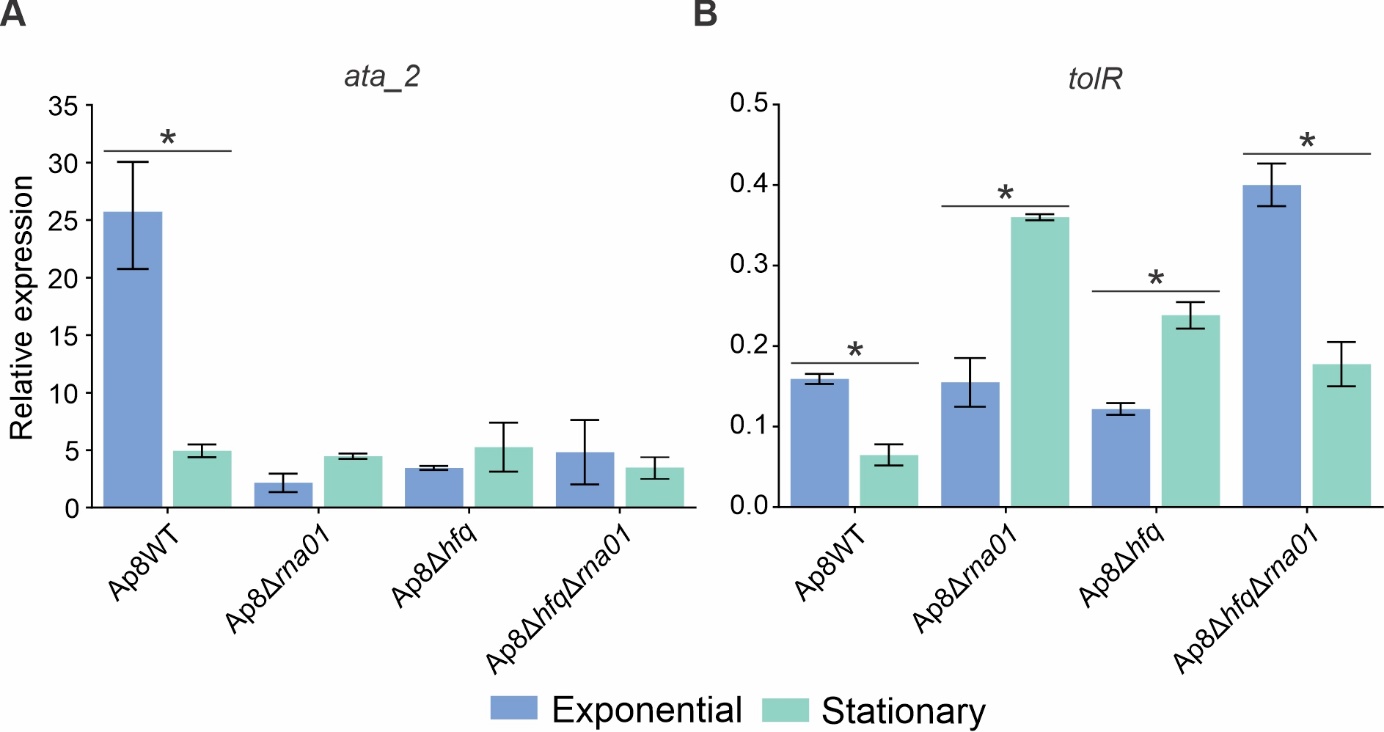


Supplementary Figure 7. Targets expression are affected by Rna01. qPCR of *ata_2* (A) and *tolR* (B) targets in exponential and stationary phase among the strains. *Significative difference by t-test (p<0.1).

Supplementary Table 3. Pairwise comparisons of the log-hank test of *G. mellonella* infection assay with *A. pleuropneumoniae* strains

| **Comparisons** | **P-Value** | **Significant?** |
| --- | --- | --- |
| Ap8WT vs. PBS | 0,000000409 | Yes |
| Ap8WT vs. Ap8∆*hfq* | 0,00000409 | Yes |
| Ap8WT vs. Ap8∆*hfq*∆*rna*01 | 0,0000915 | Yes |
| Ap8WT vs. Ap8∆*rna*01 | 0,0136 | Yes |
| Ap8∆*rna*01 vs. PBS | 0,0153 | Yes |
| Ap8∆*rna*01 vs. Ap8∆*hfq* | 0,0667 | No |
| Ap8∆*hfq*∆*rna*01 vs. PBS | 0,277 | No |
| Ap8∆*rna*01 vs. Ap8∆*hfq*∆*rna*01 | 0,288 | No |
| Ap8∆*hfq* vs. Ap8∆*hfq*∆*rna*01 | 0,520 | No |
| Ap8∆*hfq* vs. PBS | 0,317 | No |

Supplementary Table 4. Pairwise comparisons of the log-hank test of *G. mellonella* infection assay with EVs from *A. pleuropneumoniae* strains

| **Comparisons** | **P-Value** | **Significant?** |
| --- | --- | --- |
| Ap8∆*rna*01 vs. Ap8∆*hfq* | 0,00000495 | Yes |
| Ap8WT vs. Ap8∆*rna*01 | 0,0000175 | Yes |
| Ap8∆*rna*01 vs. Ap8∆*hfq*∆*rna*01 | 0,000305 | Yes |
| Ap8∆*rna*01 vs. PBS | 0,000839 | Yes |
| Ap8∆*hfq*∆*rna*01 vs. PBS | 0,619 | No |
| Ap8∆*hfq* vs. Ap8∆*hfq*∆*rna*01 | 0,592 | No |
| Ap8WT vs. PBS | 0,777 | No |
| Ap8WT vs. Ap8∆*hfq*∆*rna*01 | 0,765 | No |
| Ap8∆*hfq* vs. PBS | 0,729 | No |
| Ap8WT vs. Ap8∆*hfq* | 0,559 | No |


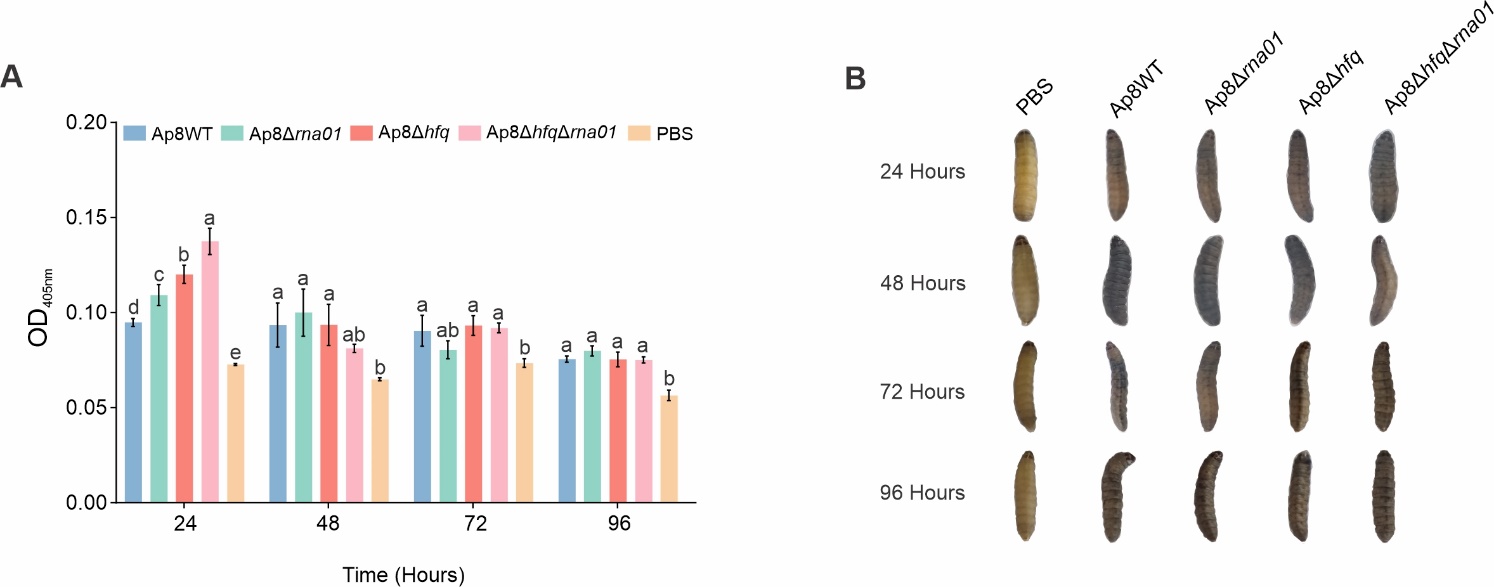


**Supplementary Figure 8.** Extracellular vesicles cause strong melanization in *G. mellonella*. **(A)** optical density of larval hemolymph post-infection. **(B)** visual observation of larval melanization through the course of the experiment. Means with different letters are significantly different by Tuckey’s test
